# Mindfulness-based interventions for non-affective psychosis: a comprehensive systematic review and meta-analysis

**DOI:** 10.1080/07853890.2022.2108551

**Published:** 2022-08-25

**Authors:** Annie Lai King Yip, Thanos Karatzias, Wai Tong Chien

**Affiliations:** School of Health and Social Care, Edinburgh Napier University, Sighthill Campus, Edinburgh, UK

**Keywords:** Mindfulness-based interventions, non-affective psychosis, effective to psychotic symptoms/affective symptoms/quality of life/mindfulness skills/insight into illness/treatment, longer-term duration, younger people

## Abstract

**Aim:**

Although mindfulness-based interventions (MBIs) are routinely used in clinical practice, a comprehensive synthesis of the effectiveness of MBIs for non-affective psychosis has yet to be conducted. The aim of the present review and meta-analysis was to investigate the effectiveness of MBIs including those with mindfulness as an active treatment component for alleviating symptoms of psychosis to inform future clinical practice.

**Methods:**

A systematic review of studies published in journals or in dissertations in CINAHL, PubMed, EMBASE, PsycINFO, CENTRAL, ISRCTN, or CNKI from January 1990 until December 2020. A total of 31 eligible studies (*n* = 2146) were included.

**Results:**

Effect-size estimates suggested that 22 independent samples (*n* = 1632) produced a statistically significant small effect for psychotic symptoms (*g* = −0.48), and with a clinically significant reduction of 50% from baseline (pooled OR: 1.84). Separate meta-analyses demonstrated small effects for affective symptoms (*g* = −0.44) and small**-**to-large positive effects for quality of life (*g* = 0.38), mindfulness skills (*g* = 0.45), and insight into illness/treatment (*g* = 1.35). The heterogeneity was high across the studies.

**Conclusion:**

Results suggest that short-term MBIs can be beneficial for non-affective psychosis. Future research is needed to test the efficacy and safety of dedicated MBIs for this population group over a longer term.

**KEY MESSAGES**
- Schizophrenia spectrum and other psychotic disorders, also known as non-affective psychosis, is the most chronic and debilitating type of psychosis, seriously affecting every aspect of a person’s life, including social, occupational, or general functioning.
- The aim of the current systematic review and meta-analysis was to investigate formerly unexamined questions regarding the clinical significance of MBIs including yoga as an increasingly utilized, conceptualized psychological intervention on overall psychotic symptoms for people with non-affective psychosis.
- No serious adverse events were reported in the studies, suggesting that MBIs may be safe interventions, while there is robust evidence to support the view that MBIs are beneficial to young people in particular.

## Introduction

Psychosis includes primary psychosis, such as bipolar disorders or depressive disorders with psychotic features, schizophrenia spectrum and other psychosis disorders; and secondary psychosis due to neurological and medical conditions. Schizophrenia spectrum and other psychotic disorders, also known as non-affective psychosis [1], is the most chronic and debilitating type of psychosis, seriously affecting every aspect of a person’s life, including social, occupational, or general functioning. In recent meta-analyses [2–4], it was suggested that psychotherapy plus treatment-as-usual (TAU) tended to be more effective than medication only. In particular, mindfulness-based interventions (MBIs) may be a useful psychotherapeutic modality for psychosis, despite concerns regarding safety issues of mindfulness practice [5,6]. Mindfulness, which is originated in ancient India, is dated back more than 2500 years and related to the philosophy of Buddhism. In 1970s, Dr. Jon Kabat-Zinn integrated mindfulness meditation, body awareness, and yoga to develop a program called mindfulness-based stress reduction for the western world. The practice emphasizes awareness of the present moment and encourages individuals to experience with acceptance rather than avoidance or control. This concept has been further adopted in various practices, such as mindfulness-based cognitive therapy, which has been labelled as a third-wave cognitive therapy treating the process of thinking and feeling rather than handling the content of psychotic symptoms [7], or acceptance and commitment therapy which aims at improving affective symptoms by facilitating psychological flexibility [8]. Currently, there are six meta-analyses on the efficacy of MBIs for people with psychosis, including affective disorders [9–14]. From the results of the current meta-analyses, MBIs seem to have beneficial effects on negative symptoms, overall psychotic symptoms, depression, and hospitalization rates, but the benefits were uncertain for positive symptoms, anxiety, and social functioning. Where MBIs were more effective in a group and appeared largely safe for treating people with psychosis. In addition, moderate short-term effects were observed in most of the above-mentioned reviews on overall psychotic symptoms. However, it is difficult to draw conclusions on the effectiveness of MBIs, as very few previous meta-analyses that included a large number of RCTs across various MBIs for people with non-affective psychosis have been conducted. MBIs can include a range of acceptance and mindfulness-based interventions, such as mindfulness-based cognitive approaches, mindfulness-based psychoeducation, or yoga. It is uncertain whether these approaches are effective for non-affective psychosis in relation to clinical outcomes, such as psychotic symptoms, and affective symptoms.

The aim of the current systematic review and meta-analysis was to investigate formerly unexamined questions regarding the clinical significance of MBIs including yoga as an increasingly utilized, conceptualized psychological intervention [15–17] on overall psychotic symptoms for people with non-affective psychosis when compared to the usual treatment and/or other psychosocial interventions. The secondary outcomes of the review included affective symptoms, mindfulness skills, socio-occupational functioning, QoL, and others (outcomes resulting from three or more studies).

## Method

**Figure 1.**
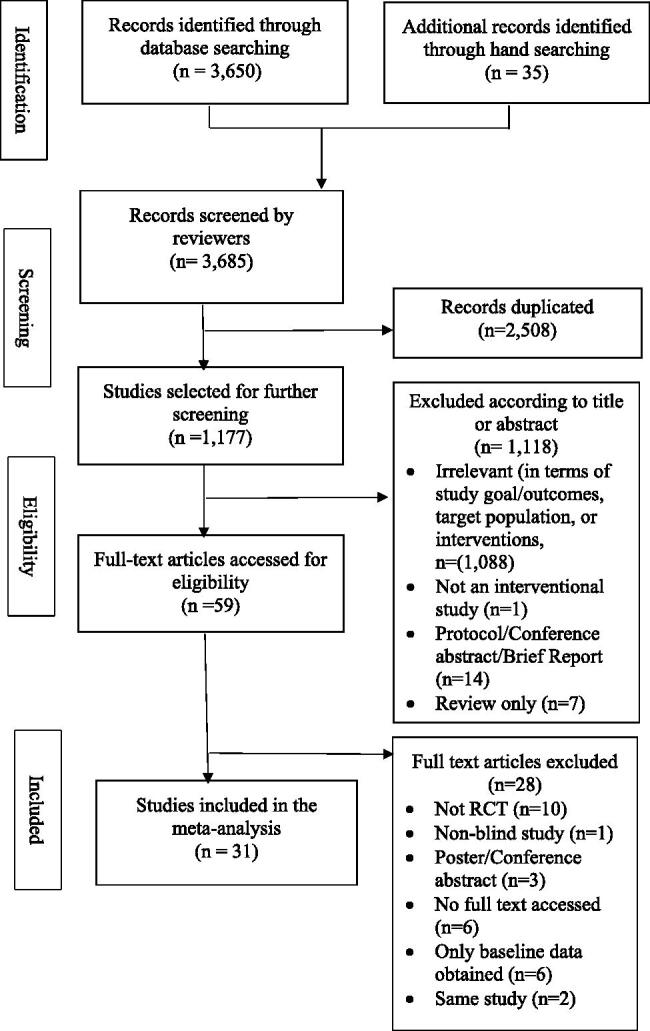
Literature search strategy (PRISMA flow diagram).

The review protocol was registered in advance with the International Prospective Register of Systematic Reviews (PROSPERO), Registration Number: CRD42017074925. This review was conducted in accordance with the Preferred Reporting Items for Systematic Reviews and Meta-analyses (PRISMA) guidelines [18] (see Figure 1).

### Inclusion and exclusion criteria

The search focussed on full reports in Chinese and English of single-blinded or open RCTs, including clustered, cross-over, and wait-list controlled trials, published between January 1990 and December 2017. Only studies that examined the effectiveness of mindfulness-based interventions on people with psychosis were included. Mindfulness processes that are mainly derived from Kabat-Zinn’s [19] and Teasdale, Williams, and Segal’s [20] mindfulness protocols were included, while interventions, such as acceptance-based mindfulness, yoga mindfulness, and compassionate meditation were also considered for the present review. Such specification is aimed at minimizing any bias induced by various mindfulness approaches. The studies that were included were those of adults in various mental health care settings, including out-patient, in-patient, day-care, and community care services, with a primary diagnosis of a psychotic disorder (as diagnosed using any recognized diagnostic criteria) as compared with another intervention, such as psychoeducation and work-related training with or without TAU, or standard care only.

Exclusion criteria included people with primary diagnoses, such as neurodevelopment disorders, neurocognitive disorders, or psychotic disorders due to another medical condition, and who had a primary substance-related or alcohol addiction and/or any clinically significant medical diseases, or with a primary diagnosis of affective disorders, such as major depression with psychotic symptoms. Also excluded were studies in which <50% of the participants had received a primary diagnosis of a non-affective psychotic disorder, and/or in which <50% of the content was mindfulness-based [19,20]. Only studies with outcome measures having high validity and inter-rater reliability, such as the Positive and Negative Syndrome Scale (PANSS) [21] were included to minimize detection/outcome bias.

### Search strategy

As recommended [22], a series of processes were used to conduct a thorough literature search: (i) searching multiple electronic databases, (ii) screening the reference lists of relevant studies, (iii) hand-searching relevant key journals, (iv) searching trial registers, and (v) contacting authors for additional information. Searches were conducted, therefore, in the following databases: CINAHL Complete, PubMed (NLM), EMBASE, PsycINFO (ProQuest), the Cochrane Library (Wiley Online Library), the International Standard Randomized-Controlled Trial Number Register (ISRCTN), Clinicaltrials.gov, the National Centre for Complementary and Alternative Medicine, the WHO International Clinical Trials Registry Platform Search Portal, the China Knowledge Network (CNKI), and the Australian New Zealand Clinical Trials Registry. The search terms included (mindfulness OR mindful* OR yoga OR meditation OR acceptance and commitment therapy AND psychotic disorders OR psychosis OR psychot* OR schizophrenia OR schizophrenia spectrum disorders) relating to the keywords of the study from all fields, free text, and Medical Subject Headings (MeSH) for search expansion from January 1990 to December 2017. The Boolean operators OR were used to separate the combinations of search terms. Filters were also used to narrow the search to specific journals, peer-reviewed articles, dissertations, and theses. A hand-search of internet search engines, such as Google Scholar for grey literature (unpublished reports or articles) was implemented for additional studies, and authors were contacted for missing or unpublished data. The process was supported by two independent reviewers (the author and WW) and overseen by TK. Screening of titles and/or abstracts of individual articles was carried out by the author to identify studies that met the inclusion criteria. Full texts of the potentially eligible studies were retrieved and independently assessed by the author and WW. Any discrepancies between the two were resolved through discussion with TK. The search was re-run in December 2020 and any new studies retrieved were included for further screening. See Supplementary Material (Appendix A) for the searching process.

### Data extraction and categorization

In keeping with published recommendations [23], a standardized extraction form was designed to extract data from the chosen studies on aspects, such as demographics, study design, population, interventions and comparators, outcome measurements, clinical outcomes, and information for the assessment of the risk of bias. Data were extracted independently by the author and SH, who resolved their disagreements through discussion, with the involvement of two supervisors (TK/WTC). If there were missing data in a study, the authors of the study were contacted and asked to provide the relevant raw data. Studies with an attrition rate of over 50% were excluded from the outcome analysis.

### Risk of bias and coding

The Cochrane risk-of-bias tool for randomized trials (RoB 2.0) [23] was used to assess the internal validity of the included studies. The author and SH independently assessed the five domains of bias, including bias arising from the randomization process, bias due to deviations from intended interventions, bias due to missing outcome data, bias in the measurement of the outcome, and bias in the selection of the reported result. Sample sizes of under 20 were suggested as small in this study [24]. The studies were categorized into Low, Some concerns, or High risk of bias according to the above five domains. A thorough examination was also carried out to check for other biases. The GRADEpro GDT approach https://gdt.gradepro.org/app/handbook/handbook.html#h.33qgws879zw was adopted to assess the overall quality of the evidence in the estimates of effect size and the quality of the clinical outcomes across the included studies.

### Statistical meta-analysis

RevMan 5.3 [25] was used to compute the statistical analysis, which included creating forest plots. The random-effects model was used to estimate the treatment effect sizes between the treatment group and the comparison groups [26]. The estimates of treatment effect could vary across studies because of real differences in the effect of the intervention [27]. In addition, outcomes from three or more studies were included in the model for a meta-analysis. Hedges’s *g* standardized mean difference (SMD) with 95% confidence intervals (CIs) was calculated [28] for each outcome within each study design. This helped to adjust for another potential bias, namely, the tendency to overestimate the effect size in small samples [29]. Cohen’s categories [29] were used, where SMD 0.2, 0.5, and 0.8 indicated small, medium, and large effects, respectively. A two-sided *p*-value was used to indicate statistical significance if the probability of the calculated mean difference was <5% (*p* < .05). Following the main analysis, separate meta-analyses were conducted to examine the effects of subtypes of MBIs for individual outcomes when data was available. A study was considered an outlier when its 95% confidence interval (CI) was outside the 95% CI of the overall mean effect size on both sizes. Outliers were identified through visual inspection of the forest plots, and repeated analyses were performed.

### Heterogeneity

Heterogeneity was explored using Cochran's *Q*, *I*^2^, and the *τ*^2^ statistics. Cochran's *Q* is the usual test statistic, which reflects systematic between-study differences. The *I*^2^ statistic is an estimate of the degree of heterogeneity. An *I*^2^ value of 0% indicates no observed heterogeneity. Values of 25, 50, and 75% are considered low, moderate, and high, respectively [30], with a value of 75% or higher being indicative of substantial heterogeneity. *τ*^2^ is the variance of the true effect sizes, calculated as part of the random effects meta-analyses. A contour-enhanced funnel plot and Egger’s statistical test for symmetry were used to examine the potential for publication bias of an outcome originating from at least 10 or more studies [31,32]. When no publication bias and between-study heterogeneity were present, the funnel plot should show a symmetrical funnel-like shape. Asymmetry in a funnel plot is potentially indicative of publication bias. Regarding Egger’s test, a *p*-value of <.10 was taken to indicate statistical evidence of asymmetry [32,33].

### Clinical significance

Since clinicians criticize SMDs as non-intuitive and difficult to interpret [31], the use of a responder is preferable, to convert the result of continuous outcomes into dichotomous treatment responses. In the present analysis, two response cut-offs (a reduction of at least 25% and 50% in the PANSS total score from the baseline) were calculated for the 18 trials [34].

## Results

### Study selection and characteristics

#### Study selection

A total of 3650 records were identified through searching the electronic databases. Another 35 records were found from a manual search. A total of 3685 studies were screened, and 1177 studies were left for further screening after 2508 duplicates were removed. Of the 1177 publications that were examined, 31 single-blinded RCTs (*n* = 2146) were included in one or more of the meta-analyses.

#### Study characteristics

Among the included studies, the number of interventions featuring yoga, a mindfulness intervention (MI)/MI plus CBT, acceptance and commitment therapy (ACT)/ACT plus cognitive-behavioural therapy (CBT), and MI with psychoeducation were 12, 11, 5, and 3, respectively. The primary outcome measures were mainly psychotic symptoms (*k* = 9), clinical functioning (*k* = 4), and affective symptoms (*k* = 3). The range of the sample size was from 19 to 342. The mean proportion of females involved in the studies was 39.7%. Thirteen studies used a passive control group as the comparison. Nine studies were carried out in European countries, eleven in Asia, seven in Asia-Pacific countries, two in Canada, and two in the United States. Ten studies were conducted in out-patient units, eight in in-patient units, three studies in both out-patient and in-patient units, and ten studies involved subjects recruited from community venues. In terms of attrition rates, 31 studies had attrition rates of 4.3–45.1% (studies with attrition rates of below 50% were considered eligible for inclusion). Attrition rates were not reported in nine studies.

Of the 31 studies, 19 did not report an ITT analysis. The percentage of people with non-affective psychosis recruited for individual studies ranged from 73 to 100%. In 24 studies, 100% of the included participants had non-affective psychosis. Only Gumley et al. [35] adopted an individual-based intervention, while a group-based format was used in the other studies. Regarding treatment durations, Manjunath et al. [36] delivered a 2-week intervention and Lopez-Navarro et al. [37,38] delivered a 26-week therapy, making these studies the shortest and the longest, respectively. In terms of hours of therapy, the interventions ranged from 8 to 40 h for each delivery; however, the duration of the therapies that were applied was not reported in the studies of Behere et al. [39], Chadwick et al. [40], Gumley et al. [35], and Yang and Zhu [41]. Nineteen studies arranged for home practice, either compulsory or non-compulsory, following guided sound-tracks or the completion of a log book. Five studies used three-arm interventions [39,42–44]. The active control varied from exercise to conventional psychoeducation, physical exercise, day care, or the practice of befriending. Four studies [42,45–47] recruited participants with mean ages of under 30, while 22 studies included participants with mean ages of 30–50 [7,35–41,43,44,48–59]. The remaining five studies included subjects with mean ages of over 50 [60–64]. Only ten studies had three to five points of assessment from baseline and one week to 24-month intervals after the interventions [7,35,36,39,42,45,46,52,60,64]. The other studies adopted pre- and post-therapy assessments.

### Summary of the findings

A synthesis of the data on outcome measures from at least three studies included data on psychotic symptoms (*k* = 22, *n* = 1632), affective symptoms (*k* = 11, *n* = 641), quality of life (QoL) (*k* = 10, *n* = 547), socio-occupational functioning (*k* = 9, *n* = 968), mindfulness skills (*k* = 8, *n* = 433), clinical functioning (*k* = 7, *n* = 365), insight into illness/treatment (*k* = 6, *n* = 736), auditory hallucinations and delusions (*k* = 5, *n* = 315), side-effects (*k* = 5, *n* = 304), client satisfaction (*k* = 3, *n* = 173), psychological flexibility (*k* = 4, *n* = 170), and emotional regulation (*k* = 3, *n* = 162). A meta-analysis on re-hospitalization was not performed because fewer than three studies with sufficient data were obtained (see Appendices B and C in Supplementary Materials).

#### Risk of bias

**Figure 2.**
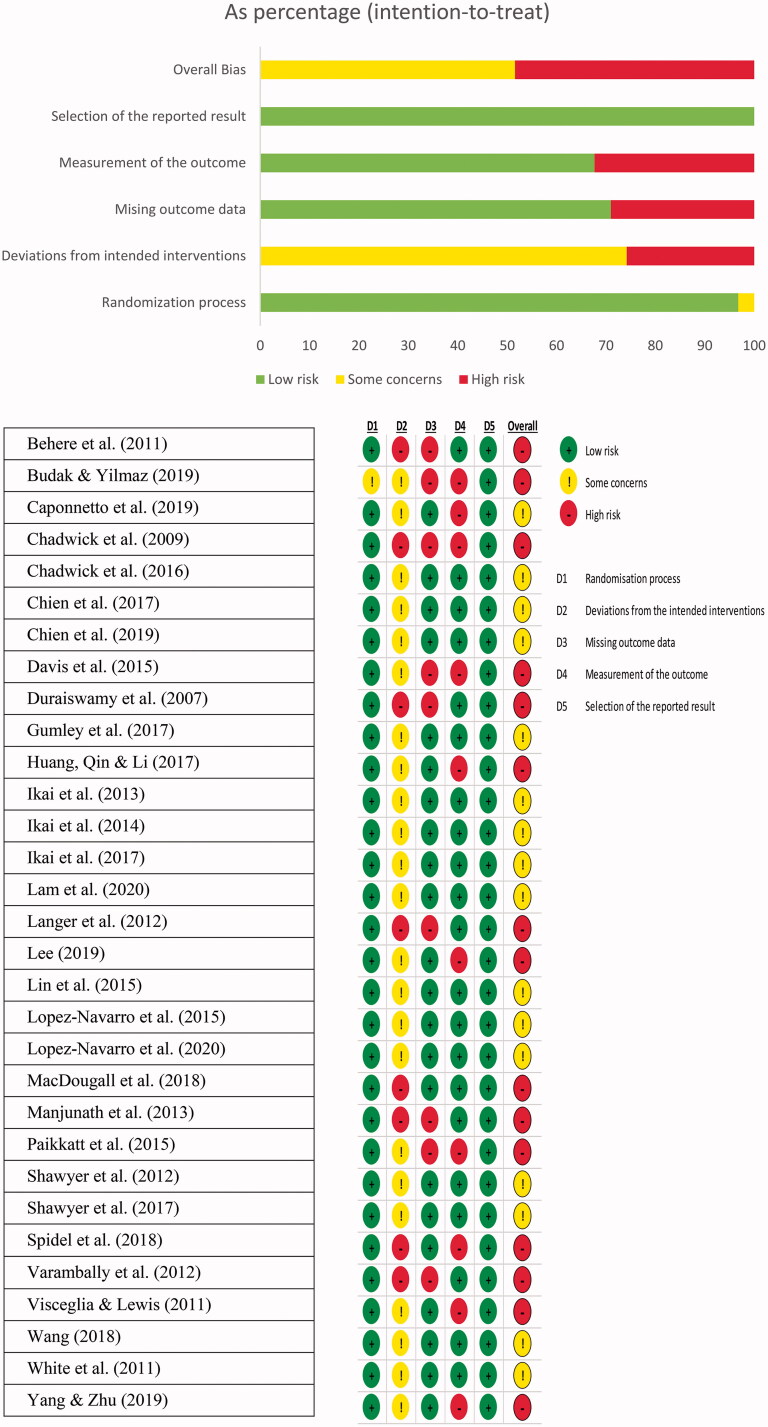
Cochrane risk of bias ratings (RoB 2.0).

All studies were judged as being of low risk of bias in terms of the randomization process, with the exception of the study which had some concerns [48]. Eight studies were deemed to be of high risk with regard to deviations from the intended interventions (25.8%), while the remaining studies presented some concerns. Nine studies were at high risk in the aspect of missing outcome data, while 22 studies (71%) presented as low risk. Ten studies were reported as being of high risk in the domain of measurement of the outcome (32.3%), while the rest of the studies were reported as being of low risk. All studies were judged as being of low risk in the selection of the reported results. Overall, 15 studies were deemed to be at high risk of bias (48.4%). The rest were judged as having some concerns in at least one domain (see Figure 2). Among all of the studies, 19 did not report/follow ITT principles in their analysis (61.3%), while 29 adopted manualized treatment protocols (93.5%) (see Table 1). Risk of bias ratings (RoB 2.0) for the individual studies can be found in Supplementary Materials (see Appendix D in Supplementary Material).

**Table 1.** Cochrane risk of bias ratings (RoB 2.0).

Study	Randomization process	Deviations from intended interventions	Missing outcome data	Measurement of the outcome	Selection of the reported results	Manualized treatment protocols	ITT principles	Overall bias
Behere et al. (2011)	Low	High	High	Low	Low	Yes	NR	High
Budak and Yilmaz (2019)	Some concerns	Some concerns	High	High	Low	Yes	NR	High
Caponnetto et al. (2019)	Low	Some concerns	Low	High	Low	Yes	NR	Some concerns
Chadwick et al. (2009)	Low	High	High	High	Low	Yes	No	High
Chadwick et al. (2016)	Low	Some concerns	Low	Low	Low	Yes	Yes	Some concerns
Chien et al. (2017)	Low	Some concerns	Low	Low	Low	Yes	Yes	Some concerns
Chien et al. (2019)	Low	Some concerns	Low	Low	Low	Yes	Yes	Some concerns
Davis et al. (2015)	Low	Some concerns	High	High	Low	Yes	No	High
Duraiswamy et al. (2007)	Low	High	High	Low	Low	Yes	NR	High
Gumley et al. (2017)	Low	Some concerns	Low	Low	Low	Yes	Yes	Some concerns
Huang et al. (2017)	Low	Some concerns	Low	High	Low	Yes	NR	High
Ikai et al. (2013)	Low	Some concerns	Low	Low	Low	Yes	Yes	Some concerns
Ikai et al. (2014)	Low	Some concerns	Low	Low	Low	Yes	NR	Some concerns
Ikai et al. (2017)	Low	Some concerns	Low	Low	Low	Yes	Yes	Some concerns
Lam et al. (2020)	Low	Some concerns	Low	Low	Low	Yes	Yes	Some concerns
Langer et al. (2012)	Low	High	High	Low	Low	Yes	NR	High
Lee (2019)	Low	Some concerns	Low	High	Low	Yes	Yes	High
Lin et al. (2015)	Low	Some concerns	Low	Low	Low	Unclear	Yes	Some concerns
Lopez-Navarro et al. (2015)	Low	Some concerns	Low	Low	Low	Yes	Yes	Some concerns
Lopez-Navarro et al. (2020)	Low	Some concerns	Low	Low	Low	Yes	Yes	Some concerns
MacDougall et al. (2018)	Low	High	Low	Low	Low	Yes	No	High
Manjunath et al. (2013)	Low	High	High	Low	Low	Yes	No	High
Paikkatt et al. (2015)	Low	Some concerns	High	High	Low	Yes	NR	High
Shawyer et al. (2012)	Low	Some concerns	Low	Low	Low	Yes	No	Some concerns
Shawyer et al. (2017)	Low	Some concerns	Low	Low	Low	Yes	Yes	Some concerns
Spidel et al. (2018)	Low	High	Low	High	Low	Yes	NR	High
Varambally et al. (2012)	Low	High	High	Low	Low	Yes	No	High
Visceglia and Lewis (2011)	Low	Some concerns	Low	High	Low	Unclear	NR	High
Wang (2018)	Low	Some concerns	Low	Low	Low	Yes	NR	Some concerns
White et al. (2011)	Low	Some concerns	Low	Low	Low	Yes	NR	Some concerns
Yang and Zhu (2019)	Low	Some concerns	Low	High	Low	Yes	NR	High

#### Quality of the evidence

The overall GRADEpro assessment of the outcomes indicated that the quality of the evidence was from “Moderate” to “Very Low”. The evidence was “Moderate” quality for the outcomes of QoL, side-effects, auditory hallucinations and delusions, psychological flexibility, client satisfaction, and mindfulness skills, while the evidence was of “Low” quality for psychotic symptoms and client satisfaction. “Very Low” ratings were accorded to the evidence for the remaining outcomes, including affective symptoms, socio-occupational functioning, insight into illness/treatment, clinical functioning, and emotion regulation (see Appendix D in Supplementary Material).

### Efficacy of the treatments

See Appendix E in Supplementary Material for the forest plots.

#### Effects on psychotic symptoms (primary outcome)

**Figure 3.**
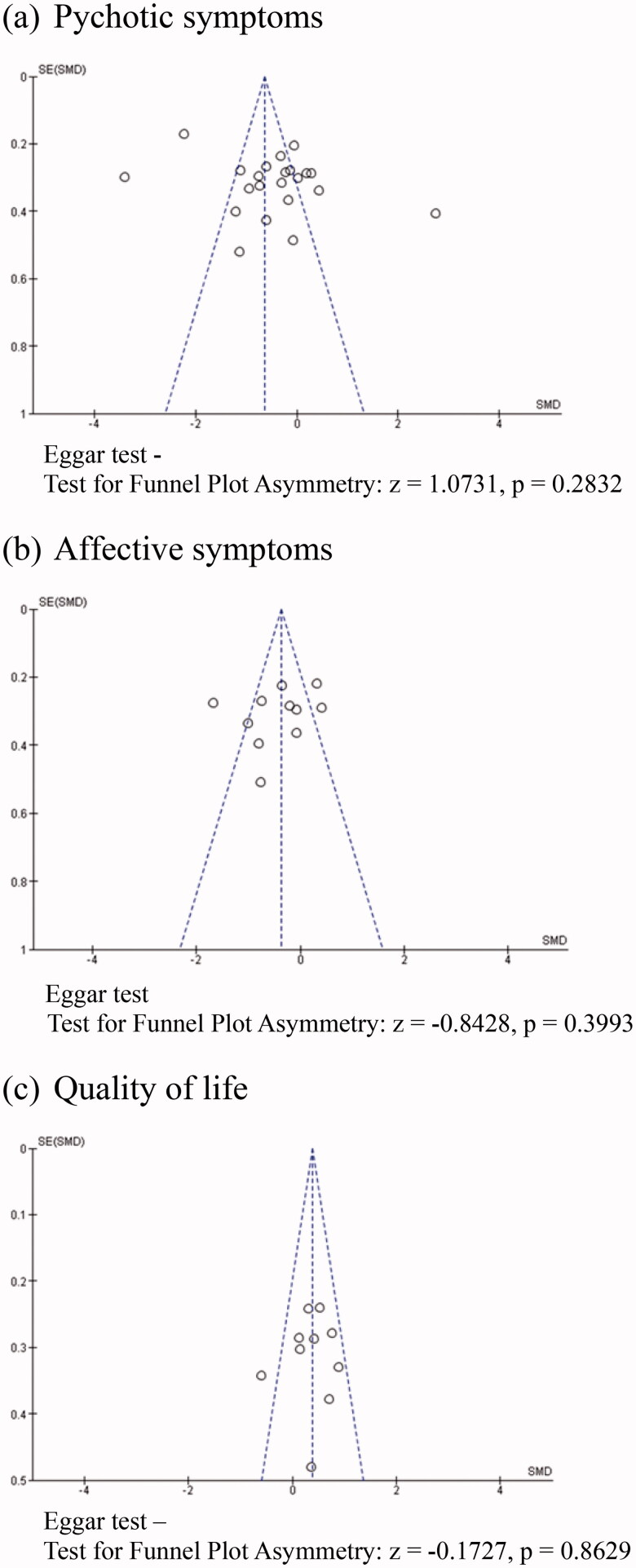
Funnel plots. (a) Psychotic symptoms. Eggar test—test for funnel plot asymmetry: *z* = 1.0731, *p* = .2832. (b) Affective symptoms. Eggar test—test for funnel plot asymmetry: *z* = −0.8428, *p* = .3993. (c) Quality of life. Eggar test—test for funnel plot asymmetry: *z* = −0.1727, *p* = .8629.

For psychotic symptoms (*k* = 22, *n* = 1632), a significant, small pooled effect size was observed (*g* = −0.48, *p* < .05). The level of heterogeneity was large (*I*^2^ = 93%), indicating a high risk of inconsistency. Three outliers were detected [42,45,64] from the funnel plot (*p* = .283) (see Figure 3). After these studies were removed from the analysis, we found a similar effect, with *g* = −0.36 (*p* < .001) and moderate heterogeneity (*I*^2^ = 56%). In a subgroup analysis (*k* = 9, *n* = 847), the results showed that there were no significant differences between those studies and the studies in which psychotic symptoms were not the primary outcome (*p* = .45), and that the level of heterogeneity remained substantial (*I*^2^ = 93%). Due to heterogeneity across the studies (*I*^2^ = 93%, *p* < .001), the therapies were classified as falling under four types, namely Yoga Therapy (Yoga), Acceptance and Commitment Therapy (ACT), Mindfulness Intervention (MI), and mindfulness-based psychoeducation (PsychoED). A significantly higher reduction in psychotic symptoms was seen with Yoga and PsychoED than with TAU and/or other psychosocial interventions, with *g* = −0.40 (*p* < .005), and *g* = −2.78 (*p* < .005), respectively. To investigate the effectiveness of interventions over time, post-intervention durations ranging from two weeks to six months were classified as short-term, while durations ranging from 18 to 24 months were classified as long-term. Since the time points for the data collection for two studies were 3 and 18 months, and 6 and 24 months, respectively, two studies were included in both the short- and long-term analyses [45,46]. The studies spanning a longer period had a large effect (*g* = −1.98, *p* < .001) and no outlier, while shorter-term studies had a small effect (*g* = −0.33, *p* < .001). The level of heterogeneity was substantial for both long- and short-term treatments. Regarding the response analysis of at least 25 and 50% from baseline, it was found that the pooled ORs were *g* = 2.87 (*p* = .003) and *g* = 1.84 (*p* = .03), respectively. The results also showed that 57.9% of clients who received a mindfulness treatment had a reduction of at least 25% in PANSS compared to the baseline, while 26.1% had a reduction of at least 50% in PANSS. By contrast, the reductions in PANSS for the control group were 27.2 and 13.1%, respectively (see Table 2). The participants in the included studies were classified into four age groups, namely, 20–50, 30–50, mean age of 30, and over 50. In the overall results, significant differences were seen between age categories. The groups aged below 50 had significantly small to high effects, *g* = −0.36 to −1.58, whereas those aged over 50 had an insignificantly small effect size of *g* = 0.49.

**Table 2.** Percentage PANSS-derived responder rate for at least 25 and 50% PANSS reduction from baseline.

	25% PANSS reduction						50% PANSS reduction					
	Experimental				Control		Experimental				Control	
	*N*	No of responders	Response rate	*N*	No of responders	Response rate	*N*	No of responders	Response rate	*N*	No of responders	Response rate
Behere et al. (2011)	27	10	(37.0)	22	6	(27.3)	27	2	(7.4)	22	1	(4.5)
Chien et al. (2017)	111	74	(66.7)	112	7	(6.3)	111	14	(12.6)	112	1	(0.9)
Chien et al. (2019)	56	55	(98.2)	56	1	(1.8)	56	30	(53.6)	56	0	(0.0)
Duraiswamy et al. (2007)	21	17	(81.0)	20	11	(55.0)	21	12	(57.1)	20	7	(35.0)
Ikai et al. (2013)	25	7	(28.0)	24	6	(25.0)	25	2	(8.0)	24	2	(8.3)
Ikai et al. (2014)	25	6	(24.0)	25	7	(28.0)	25	2	(8.0)	25	4	(16.0)
Ikai et al. (2017)	24	6	(25.0)	25	8	(32.0)	24	1	(4.2)	25	3	(12.0)
Lee (2019)	20	4	(20.0)	30	4	(13.3)	20	1	(5.0)	30	1	(3.3)
Lin et al. (2015)	39	17	(43.6)	18	6	(33.3)	39	12	(30.8)	18	4	(22.2)
Lopez-Navarro et al. (2015)	22	6	(27.3)	22	5	(22.7)	22	1	(4.5)	22	2	(9.1)
Lopez-Navarro et al. (2020)	26	12	(46.2)	26	9	(34.6)	26	6	(23.1)	26	4	(15.4)
Manjunath et al. (2013)	35	34	(97.1)	25	23	(92.0)	35	25	(71.4)	25	13	(52.0)
Paikkatt et al. (2015)	15	15	(100.0)	15	14	(93.3)	15	15	(100.0)	15	10	(66.7)
Shawyer et al. (2012)	18	8	(44.4)	18	7	(38.9)	18	3	(16.7)	18	3	(16.7)
Shawyer et al. (2017)	41	15	(36.6)	36	11	(30.6)	41	5	(12.2)	36	4	(11.1)
Varambally et al. (2012)	39	29	(74.4)	34	15	(44.1)	39	12	(30.8)	34	10	(29.4)
Visceglia and Lewis (2011)	10	8	(80.0)	8	1	(12.5)	10	4	(40.0)	8	0	(0.0)
White et al. (2011)	14	6	(42.9)	10	2	(20.0)	14	1	(7.1)	10	0	(0.0)
Overall	568	329	(57.9)	526	143	(27.2)	568	148	(26.1)	526	69	(13.1)
Odd ratio	2.87 [1.45, 5.68]						1.84 [1.06, 3.20]					

#### Effects on secondary outcomes

Based on 11 studies (*n* = 641), we found that MBIs had a significant, small effect size on affective symptoms, with *g* = −0.44 (*p* = .03). The level of heterogeneity was high (*I*^2^ = 80%). One outlier was found in the funnel plot [42] (*p* = .399) (see Figure 3) and after that study was excluded, the effect size was *g* = −0.29 (*p* = .01), indicating a significant impact on the overall effect size. A subgroup analysis further showed that significant differences were not due to the type of intervention (*I*^2^ = 0.00%, *p* = .46). The only exception was for the ACT, which resulted in a significant reduction in affective symptoms compared to the control groups, with a significant moderate effect size of *g* = −0.64 (*p* = .01).

*Other secondary outcomes* (see Appendices F and G in Supplementary Materials).

## Discussion

### Main findings

See Appendix H in Supplementary Material for a summary of the findings.

A total of 22 studies reported outcomes on psychotic symptoms and included mostly participants with a diagnosis of non-affective psychosis. In contrast to the finding of Li et al. [12] and Sabe et al. [14]. It was found that the effect was small on negative symptoms (*g* = −0.36). Both studies included mindful exercises, such as yoga, tai-chi, or qi-gong as a comparison to control groups. However, tai-chi is defined as an ancient Chinese discipline of meditative movements practised as a system of exercises, and is actually a form of Chinese boxing, while qi-gong is an ancient Chinese healing art involving meditation, controlled breathing, and movement exercises [65]. By contrast, the state of mindfulness refers to the practice of maintaining a non-judgemental state of heightened or complete awareness of one's thoughts, emotions, or experiences on a moment-to-moment basis [66]. Both tai-chi and qi-gong require practitioners to be disciplined and self-controlled to perform structured, standardized physical exercises as an ultimate goal without addressing the psychological changes. This conflicts with a non-judgemental relinquishing of awareness at the present moment that is the hallmark of mindfulness practice. Conceptually, yoga presents as a multifaceted intervention with mindfulness/meditation, breath regulation, and relaxation as essential treatment components that support distress tolerance for psychological benefit. Tai-chi and qi-gong only focus on physical manipulation [15–17]. As such, the pooled results yielded regarding the effectiveness of MBIs may not be compromised [12,14].

The findings from the current study are consistent with the findings of Louise et al. [13] suggesting that the MBIs demonstrated a small benefit for overall psychotic symptoms. In an evaluation of the efficacy of 10 RCT studies (*n* = 624, mean age range: 26–42) on third-wave interventions, which address the process of thinking and feeling rather than handling the content of the illness itself (group-based MBIs, individual-based ACT, and group-based compassion-focussed therapy) for people with psychosis, a small but significant effect (*g* = 0.29) was demonstrated for the primary outcome (psychotic symptoms). The authors reported that studies of MBIs in a group format had larger effects (*g* = 0.46) on psychotic symptoms than individual-based ACT (*g* = 0.08), although the interventions that were used were of marked heterogeneity. In the present meta-analysis, there was only one study in an individual format, and no analysis was done between group- and individual-based interventions. However, the findings suggested that a longer-term treatment duration (24–26 h) is more beneficial for psychotic symptoms than short-term interventions. Such finding echoed Louie et al.’s [13] meta-analysis that longer interventions showed a larger effect (*β* = 0.0284). In contrast to the findings of Jansen et al. [10] [16 RCT studies, *n* = 1268, mean age range: 23.8–46.8, mean years since the onset of psychosis: 8.03 (5.21)], both overall symptomatology and hospitalization rates at the end-point, right after the intervention and follow-ups, resulted in moderate to large effect sizes in both the short and long-terms of from 3 to 24 months. Cramer et al.’s [9] findings of eight RCTs (4 RCT MBIs; 4 RCT ACTs, *n* = 434, mean age range: 25.6–41.6; mean years since the onset of psychosis: 2.6–17.7) also suggested a moderate effect on the overall psychotic symptoms in a longer-term treatment and hospitalization rates of the intervention groups but no significant effect on negative symptoms when compared to the control groups. In contrast to Khoury et al.’s [11] study, which included 13 studies of people with psychotic disorders or schizophrenia-spectrum disorders (7 RCTs and 6 non-RCTs, *n* = 468, mean age range: 26–42) in their meta-analysis. They concluded that MBIs, such as ACT, were moderately effective in treating negative symptoms (*g* = 0.56). However, overall psychotic symptoms were not examined and six studies included were not RCTs. In contrast to the findings of Jansen et al. [10], the results showed significant large effects for overall psychotic symptoms (*g* = 0.8) and significant small effects on negative symptoms (*g* = 0.24). On the other hand, subgroup analyses found significant differences between the MBIs and ACT for overall psychotic symptoms, in the favour of mindfulness interventions with small to large effect sizes [9,10,13]. This was not in line with the findings from the current study, which was not significant. However, studies included both affective and non-affective psychosis [9,10,13], which had different inclusion criteria from the current study, and Jansen et al. [10] included data probably coming from the same study [67–69] that might enlarge the true effect. A high degree of heterogeneity was identified across the studies with low-quality evidence and caution should be shown when concluding these findings. In the present study, subgroup analyses found that both PsychoED and Yoga resulted in significant reductions in psychotic symptoms. The effect of Yoga was comparable to that found in previous meta-analyses [12,14]. The benefit was greater for people with psychosis under the age of 50.

Regarding the efficacy of MBIs in relation to affective symptoms, the results showed a slight association, in line with the findings of Jansen et al. [10] (*g* = 0.47), Khoury et al. [11] (*g* = 0.20), and Louie et al. [13] (*g* = 0.39), but in contrast to that of Cramer et al.’s [9] study, which showed no association. In this meta-analysis, it was found that ACT was moderately beneficial in alleviating affective symptoms and had greater effects than other mindfulness interventions, in line with the findings of Jansen et al. [10] (*g* = −0.63). Regarding social functioning and quality of life (QoL), the findings of the current review are in contrast to Jansen et al.’s meta-analyses on QoL and social functioning [10], which reported no significant effect and significant small effect, respectively (*g* = −0.43; *g* = −0.43). The difference may relate to seven studies on yoga interventions included for the analysis of QoL in the present review, while Jansen et al. [10] had different inclusion criteria. Similarly, a significant small effect size in favour of MBIs was observed for mindfulness skills (*g* = 0.45), which was in contrast to the findings of moderate effects in Jansen et al. [10] (*g* = 0.51) and Louise et al. [13] (*g* = 0.56). Louise et al. [13] also included both affective and non-affective psychosis in their study. In the present subgroup analyses, we found that MI and PsychoED showed a significant association of the treatment effect of mindfulness skills (*g* = 0.41 and *g* = 0.58, respectively). This outcome resulted in the lowest heterogeneity among the different outcomes with very low-quality evidence. A possible explanation for the lowest heterogeneity may be that the intervention protocol of these included studies originated from Mindfulness-based Stress Reduction (MBSR) [66]. The findings also showed that MBIs produced significantly large effects on insight into illness/treatment (*g* = 1.35), and PsychoED (*g* = 1.28) and Yoga (*g* = 2.92) had larger effects than other interventions. However, caution should be taken when only one to two studies were included in the analysis.

### Safety

The present meta-analysis found that there were 20 included studies that did not assess participants for their suitability of doing mindfulness practice, nor reported any adverse events, while four studies reported none but without prior study assessment on the suitability. In addition, without initial assessment, there were five included studies that reported one to two mild adverse events, such as hospitalization, unwanted experience during mindfulness practice, and one study reported two serious adverse events (both deaths) but were judged to be unrelated to the study by the Trial Steering Committee. However, only two studies included the pre-study assessment in the recruitment procedure for the suitability of mindfulness practice and reported no adverse events at the end of the study. After all, none of the included studies reported a regular examination of the safety related to mindfulness practice. Similarly, in the previous meta-analyses, there were only two studies examining safety as an outcome measure and also reported no serious adverse events or unrelated adverse events to the study [9,10].

Conclusively, the findings of the current study showed a small to large effect on affective symptoms, overall psychotic symptoms, quality of life, mindfulness skills and insight into illness/treatment, and with mindfulness favoured over acceptance with regard to long-term effects, in particular for young people. On the other hand, attention should be drawn to the small effects of yoga on overall psychotic symptoms and quality of life, and yoga may be an important component to be considered in psychological interventions. Although ACT seemed to be beneficial and superior to the mindfulness approach in alleviating affective symptoms, the results were inconclusive because less than half of the reviewed studies reported significant positive results and some did not have large samples, affecting the validity of the pooled results. Specifically, the effectiveness of MBIs in people with non-affective psychosis is uncertain due to the high heterogeneity of the samples recruited in different studies, and safety is also a concern.

### Implications for clinical practice and future research

MBIs have been criticized as having the potential to exacerbate psychosis, that is, to increase the severity of hallucinations [5,6]. In this study, only minimal of the included studies reported any adverse events. However, more RCTs are needed to assess harm by applying a recognized approach, such as a standardized recruitment protocol, a reporting system of serious adverse/adverse events, or hospital admission [70]. The results are encouraging and suggest that MBIs are effective at reducing psychotic symptoms, particularly in the form of long-term treatment for younger age groups. However, it is crucial to consider the nature of the mindfulness interventions included in this meta-analysis, as well as their varied content. The substantial heterogeneity in effect sizes found in the current study may relate to the diversity of treatment approaches. As such, it is difficult to conclude which individual treatment approach is to be recommended, because the respective focus of MI, ACT, Yoga, and PsychoED is different, such as changing relationships with thoughts, cultivating more compassion and acceptance, mindful stretching to facilitate a mind-body connection and openness to distress, and psychoeducation in the course of the illness. However, when MI was combined with Yoga and/or ACT and/or PsychoED, the correlation with the clinical outcomes was in the majority of cases higher than the one that resulted from mindfulness alone. These findings echoed those of a meta-analysis [11] that found that other mindfulness strategies might act as complementary strategies to optimize the clinical effects of mindfulness. Nevertheless, the present results provide only preliminary support for the MBIs and do not suggest the proportion of MI, ACT, Yoga, and PsychoED that should be included in therapy. Therefore, a tailor-made therapy including a focus on the aforementioned treatment approaches for an integrated treatment might result in the optimization of the clinical outcomes without overwhelming clients with treatment goals and strategies. Further research is required to test the efficacy and safety of a tailor-made intervention over a longer-term duration for several outcomes in people with psychosis. In addition, the effects between pharmacological treatments and MBIs as add-ons to pharmacological treatment should be studied.

### Strengths and weaknesses

The included studies were single-blinded RCTs, and the majority exhibited a low risk of selection bias, detection bias, and publication bias. Manualized treatment protocols were also used in most studies. Planned analytical procedures followed PRISMA guidelines, including studies from Asia, Europe, and western countries, which allowed the results to be applicable across different cultures. To minimize the effects of different interventions, only studies in which mindfulness components made up at least 50% were included in the analysis. This approach allowed the results to be reasonably comparable across MI, ACT, Yoga, and PsychoED interventions. The evidence on efficacy was robust against potential bias in several analyses of outcomes, while the results combining the GRADEpro ratings and the importance of effect sizes were described.

More than 60% of the included studies did not apply/report ITT principles that might affect the true effect of randomization. On the other hand, it is possible that the small number of RCTs in each subgroup analysis limited the presentation of the true effects. In the GRADEpro assessment subgroup, among all individual outcomes in more than 50% of the studies that were included, at least one high risk of bias was found. This increased the overall risk of bias and resulted in seven outcomes out of twelve assessed as being of “Low” to “Very Low” quality. There were high levels of heterogeneity for most outcomes due to the small number of RCTs. In addition, the interpretations of the overall effects might have been biased due to the variability in the focus of different treatment approaches, subject recruitment from different settings, and varied control group conditions.

## Conclusions

Overall, this systematic review and meta-analysis suggest that MBIs can reduce psychotic symptoms and have positive effects on affective symptoms, quality of life, mindfulness skills, and insight into illness/treatment. An intervention of over 18 months in duration is comparatively more effective at reducing psychotic symptoms than those of shorter duration. Nonetheless, substantial heterogeneity across outcomes indicated inconsistency in the included studies. No serious adverse events were reported in the studies, suggesting that MBIs may be safe interventions, while there is robust evidence to support the view that MBIs are beneficial to young people in particular.

## Supplementary Material

Supplemental Material

## Data Availability

The data that support the findings of this study are available on request from the corresponding author. The data are not publicly available due to privacy or ethical restrictions.
