# Supplementary material for: Mindfulness-based interventions for non-affective psychosis: a comprehensive systematic review and meta-analysis
Source: Ann Med. 2022 Aug 25;54(1):2340–53. doi: 10.1080/07853890.2022.2108551 (PMC9423825; doi:10.1080/07853890.2022.2108551)
Supplement: Supplemental Material [file IANN_A_2108551_SM8540.pdf]

Appendix G Effect sizes of outcome measures for included studies

Outcome (number of studies)	Studies included	N	Outcome measures (✓= primary)	Effect Size (g)	95% CI	Quality (GRADEpro)
Psychotic symptoms (22)	Behere et al. (2011)	66	PANSS ✓	0.19	[-0.37 , 0.70]	Low
	Caponnetto et al. (2019)	30	PANSS	-0.17	[-0.89 , 0.55]	
	Chien et al. (2017)	342	PANSS ✓	-2.23	[-2.56 , -1.89]	
	Chien et al. (2019)	168	PANSS	-3.39	[-3.97 , -2.81]	
	Duraiswamy et al. (2007)	61	PANSS ✓	-0.73	[-1.37 , -0.10]	
	Huang, Qin & Li (2017)	60	BPRS	-1.12	[-1.67 , -0.57]	
	Ikai et al. (2013)	49	PANSS	-0.75	[-1.33 , -0.17]	
	Ikai et al. (2014)	50	PANSS	-0.23	[-0.78 , 0.33]	
	Ikai et al. (2017)	56	PANSS	0.28	[-0.28 , 0.85]	
	Lee (2019)	50	CMV-PANSS	2.74	[1.94 , 3.53]	
	Lin et al. (2015)	124	PANSS	-0.30	[-0.92 , 0.32]	
	Lopez-Navarro et al. (2015)	44	PANSS	0.01	[-0.58 , 0.60]	
	Lopez-Navarro et al. (2020)	44	PANSS	-0.13	[-0.67 , 0.41]	
	MacDougall et al. (2018)	17	SAPS; SANS	-0.08	[-1.04 , 0.87]	
	Manjunath et al. (2013)	88	PANSS ✓	-0.61	[-1.14 , -0.09]	
	Paikkatt et al. (2015)	30	PANSS ✓	-1.20	[-1.99 , -0.42]	
	Shawyer et al. (2012)	43	PANSS ✓	0.43	[-0.23 , 1.09]	
	Shawyer et al. (2017)	96	PANSS	-0.05	[-0.45 , 0.35]	
	Spidel et al. (2018)	50	BPRS-E	-0.94	[-1.60 , -0.29]	
	Varambally et al. (2019)	119	PANSS ✓	-0.32	[-0.79 , -0.14]	
	Visceglia & Lewis (2011)	18	PANSS ✓	-1.13	[-2.14 , -0.11]	
	White el at. (2011)	27	PANSS✓	-0.61	[-1.44 , 0.23]	
Affective symptoms (11)	Caponnetto et al. (2019)	30	PSS✓	-0.08	[-0.80 , 0.63]	Very Low
	Chadwick et al. (2016)	108	HADS	-0.36	[-0.80 , 0.09]	
	Gumley et al. (2017)	29	CDSS ✓; BDI ✓	-0.21	[-0.76 , 0.35]	
	Lam et al. (2020)	46	DASS-21	-0.08	[-0.66 , 0.50]	
	Lee (2019)	50	BDI-II	0.41	[-0.17 , 0.98]	
	Lin et al. (2015)	124	CDS	0.32	[-0.11 , 0.76]	
	MacDougall et al. (2018)	17	POMS	-0.77	[-1.76 , 0.23]	
	Manjunath et al. (2013)	88	HDRS	-0.74	[-1.27 , -0.21]	
	Spidel et al. (2018)	50	GAD-7	-1.01	[-1.67 , -0.35]	
	White et al. (2011)	27	HADS ✓	-0.80	[-1.57 , -0.03]	
	Yang & Zhu (2019)	72	HAMA, HAMD	-1.68	[-2.23 , -1.14]	
Quality of life (10)	Caponnetto et al. (2019)	30	EuroQol VAS	0.70	[-0.04 , 1.44]	Moderate
	Duraiswamy et al. (2007)	61	WHOQL-BREF	0.89	[0.24 , 1.53]	
	Ikai et al. (2013)	49	EQ-5D	0.13	[-0.43 , 0.69]	
	Ikai et al. (2014)	50	EQ-5D	0.42	[-0.14 , 0.98]	
	Ikai et al. (2017)	56	EQ-5D	0.77	[0.22 , 1.31]	
	Lin et al. (2015)	124	QOL (SF-36)	0.31	[-0.16 , 0.78]	
	Lopez-Navarro et al. (2015)	44	WHOQOL-BREF ✓	0.14	[-0.45 , 0.73]	
	Shawyer et al. (2012)	43	QOL-LESQ	-0.60	[-1.27 , 0.07]	
	Visceglia & Lewis (2011)	18	WHOQOL-BREF	0.37	[-0.57 , 1.31]	
	Yang & Zhu (2019)	72	WHOQOL-BREF	0.53	[0.06 , 1.00]	

For Peer Review Only

Appendix I Effect sizes of outcome measures for included studies (Cont'd)

Outcome (number of studies)	Studies included	N	Outcome measures (✓=primary)	Effect size (*g*)	95% CI	Quality (GRADEpro)
Socio-occupational Functioning (9)	Behere et al. (2011)	66	SOFS	0.07	[-0.49 , 0.63]	Very low
	Chien et al. (2017)	342	SLOF	2.90	[2.52 , 3.28]	
	Chien et al. (2019)	168	SLOF✓	2.90	[2.37 , 3.44]	
	Duraiswamy et al. (2007)	61	SOFS	-0.48	[-1.10 , 0.15]	
	Ikai et al. (2017)	56	GAF	0.26	[-0.26 , 0.79]	
	MacDougall et al. (2018)	17	SFS	0.16	[-0.80 , 1.11]	
	Shawyer et al. (2012)	43	GAF	-0.51	[-1.18 , 0.15]	
	Shawyer et al. (2017)	96	SFS	-0.02	[-0.42 , 0.38]	
	Varambally et al. (2012)	119	SOFS	-0.15	[-0.61 , 0.31]	
Mindfulness skills (8)	Chien et al. (2019)	168	FFMQ	0.77	[0.38 , 1.15]	Moderate
	Gumley et al. (2017)	29	KIMS	0.29	[-0.44 , 1.03]	
	Lam et al. (2020)	46	FFMQ-SF	0.29	[-0.30 , 0.87]	
	Lee (2019)	50	MAAS✓	-0.01	[-0.57 , 0.56]	
	Lopez-Navarro et al. (2015)	44	MAAS	0.20	[-0.39 , 0.79]	
	Lopez-Navarro et al. (2020)	52	MAAS	0.61	[0.06 , 1.17]	
	MacDougall et al. (2018)	17	KIMS	0.35	[-0.61 , 1.31]	
	White et al. (2011)	27	KIMS	0.91	[0.11 , 1.71]	
Clinical functioning (7)	Chadwick et al. (2009)	18	CORE✓	-0.53	[-1.47 , 0.41]	Very low
	Chadwick et al. (2016)	82	CORE-OM ✓	-0.16	[-0.60 , 0.27]	
	Huang, Qin & Li (2017)	60	NOSIE	7.80	[6.27 , 9.33]	
	Ikai et al. (2013)	49	FACT-Sz	0.96	[0.36 , 1.55]	
	Ikai et al. (2014)	50	FACT-Sz	-0.05	[-0.61 , 0.50]	
	Langer et al. (2012)	18	CGI-SCH ✓	-0.63	[-1.60 , 0.35]	
	Manjunath et al. (2013)	88	CGI-S ✓	-1.20	[-1.76 , -0.64]	
Insight into illness/treatment (6)	Budak & Yilmaz (2019)	50	BIS✓	2.92	[2.10 , 3.73]	Very low
	Chien et al. (2017)	342	ITAQ	1.41	[1.11 , 1.70]	
	Chien et al. (2019)	168	ITAQ	1.09	[0.69 , 1.49]	
	Huang, Qin & Li (2017)	60	ITAQ ✓	3.51	[2.69 , 4.34]	
	Shawyer et al. (2012)	43	IS	0.30	[-0.35 , 0.95]	
	Yang & Zhu (2019)	72	SSMI-C✓	-0.89	[-1.37 , -0.40]	
Auditory hallucination and delusion (5)	Chadwick et al. (2009)	22	PSYRATS	-0.25	[-1.18 , 0.68]	Moderate
	Chadwick et al. (2016)	108	PSTRATS-AHS	0.34	[-0.11 , 0.78]	
	Lam et al. (2020)	46	C-PSTRATS	-0.43	[-1.02 , 0.16]	
	Shawyer et al. (2012)	43	PSYRATS	0.15	[-0.51 , 0.80]	
	Shawyer et al. (2017)	96	PSYRATS	-0.05	[-0.45 , 0.35]	
Side effects (5)	Duraiswamy et al. (2007)	61	SAS	-0.32	[-0.94 , 0.29]	Moderate
	Ikai et al. (2013)	49	DIEPSS	-0.04	[-0.60 , 0.52]	
	Ikai et al. (2014)	50	DIEPSS	-0.63	[-1.20 , -0.06]	
	Ikai et al. (2017)	56	DIEPSS	0.64	[0.07 , 1.22]	
	Manjunath et al. (2013)	88	SAS	-0.09	[-0.60 , 0.43]	

For Peer Review Only

Appendix I Effect sizes of outcome measures for included studies (Cont'd)

Outcome (number of studies)	Studies included	N	Outcome measures (✓= primary)	Effect Size (*g*)	95% CI	Quality (GRADEpro)
Psychological flexibility (4)	Gumley et al. (2017)	29	AAQ II	-0.34	[-1.07 , 0.40]	Moderate
	Langer et al. (2012)	18	AAQ II	-0.40	[-1.36 , 0.56]	
	Shawyer et al. (2017)	96	AAQ II	-0.11	[-0.57 , 0.35]	
	White et al .(2011)	27	AAQ II	0.41	[-0.36 , 1.17]	
Client satisfaction (3)	Davis et al. (2015)	34	CSQ-8	0.03	[-0.67 , 0.74]	Moderate
	Shawyer et al. (2012)	43	CSQ-8	0.18	[-0.48 , 0.83]	
	Shawyer et al. (2017)	96	CSQ-8	0.64	[0.23 , 1.05]	
Emotional Regulation (3)	Behere et al. (2011)	66	TRAC✓	0.08	[-0.49 , 0.64]	Very low
	Lam et al. (2020)	46	ERQ✓	0.15	[-0.45 , 0.74]	
	Spidel et al. (2018)	50	CERQ✓	0.34	[-0.29 , 0.96]	

AAQII, Acceptance and Action Scale; BDI, Beck Depression Inventory; BIS, Birchwood Insight Scale; BPRS, The Brief Psychiatric Rating Scale; BPRS-E, The Brief Psychiatric Rating Scale-Expanded; CDS, Calgary Depression Scale; CDSS, Calgary Depression Scale for Schizophrenia; CERQ, Cognitive Emotion Regulation Questionnaire; CMV-PANSS, Chinese Mandarin version of the Positive and Negative Syndrome Scale; CGIS, The Clinical Global Impression Severity; CGI-SCH, Clinical Global Impression-Schizophrenia Scale; CORE, Clinical Outcomes in Routine Evaluation; CORE-OM, Clinical Outcomes in Routine Evaluation; CSQ-8, The Client Satisfaction Questionnaire; DASS-21, Depression Anxiety Stress Scale; DIEPSS, The Drug Induced Extrapyramidal Symptoms Scale; EQ-5D, The EUroQol-5 dimensions; ERQ, Emotion Regulation Questionnaire; EuroQoL VAS, EuroQoL Visual Analogue Scale; FACT-Sz, the Functional Assessment for Comprehensive Treatment of Schizophrenia; FFMQ, Five Facet Mindfulness Questionnaire; FFMQ-SF, Five Facet Mindfulness Questionnaire-Short Form; GAD-7, The Generalized Anxiety Disorder Scale-7; GAF, Global Assessment of Functioning; HADS, Hospital Anxiety and Depression Scale; HAMA, Hamilton Rating Scale for Anxiety; HAMD, Hamilton Rating Scale for Depression; HDRS, Hamilton Depression Rating Scale; IS, The Insight Scale; ITAQ, Insight and Treatment Attitudes Questionnaire; KIMS, Kentucky Inventory of Mindfulness Skills; MAAS, Mindfulness Attention Awareness Scale; NOISE, Nurses Observation Scale For Inpatient Evaluation; PANSS, The Positive and Negative Syndrome Scale; POMS, The Profile of Mood States; PSS, The Perceived Stress Scale; PSYRATS, Psychiatric Symptom Rating; PSYRATS-AHS, Psychiatric Symptom Rating Scale-Auditory Hallucinations Scale; QOL (SF-36), The Quality of Life – Short-form 36; QOL-LESQ, The Quality of Life Enjoyment and Satisfaction Questionnaire; SANS, Scale for Assessment of Negative Symptoms; SAPS, Scale for Assesssment of Positive Symptoms; SAS, Simpson angus scale for extrapyramidal side effects; SFS, Social Functioning Scale; SLOF, Specific Levels of Functioning Scale; SOFS, Socio-Occupational Functioning Scale; SSMI-C, Stigma Scale for Mental Illness-Chinese; TRAC, TRENDS Accuracy Score; WHOQOL-BREF, World Health Organization Quality of Life.

For Peer Review Only

Appendix A Searching process

Steps	Search Term<br>Inclusion criteria:<br>Period: 1990-2020<br>All fields<br>Free text: mindfulness OR mindful* OR yoga OR meditation OR “acceptance and commitment therapy” AND psychotic disorders OR psychosis OR psychot* OR psychos* OR schizophrenia OR schizophrenia spectrum disorders<br>MeSH: psychotic disorders; mindfulness<br>MeSH and free text<br>RCT and full text	Number of articles retrieved
	**PubMed (NLM)**	
S1	mindfulness OR mindful* OR yoga OR meditation OR “acceptance and commitment therapy” AND psychotic disorders OR psychosis OR psychot* OR psychos* OR schizophrenia OR schizophrenia spectrum disorders	791,124
S2<br>Free text	(("Schizophrenia Spectrum and Other Psychotic Disorders"[MeSH Terms]) NOT ("Psychoses, Substance-Induced"[MeSH Terms])) AND ("Mindfulness"[MeSH Terms])	69
S3	MeSH OR free text	4316
S4	RCT	
S5	RCT and full text (1990-2020)	611
	**CINAHL Complete (via EbscoHost)**	
S1<br>Free text	(mindfulness OR mindful* OR yoga OR meditation OR “acceptance and commitment therapy”) AND (“psychotic disorders” OR psychosis OR psychot* OR psychos* OR schizophrenia OR “schizophrenia spectrum disorders”)	6153
S2	(MH "Schizophrenia+") OR (MH "Psychotic Disorders+")	132,876
S3	(MH "Mindfulness") OR (MH "Mind Body Techniques")	49,670
S4	S2 AND S3	1,794
S5	S1 OR S4	7,755
S6	RCT and full text (1990-2020)	198

For Peer Review Only

	**EMBASE**	
S1 Free text	mindfulness OR mindful* OR yoga OR meditation OR "acceptance and commitment therapy" AND "psychotic disorders" OR psychosis OR psychot* OR psychos* OR schizophrenia OR "schizophrenia spectrum disorders"	7,079
S2	'schizophrenia spectrum disorder'/exp OR 'psychosis'/exp	320,776
S3	'mindfulness'/exp	10826
S4	S2 AND S3	336
S5	S1 OR S4	7102
S6	RCT and full text (1990-2020)	950
	**PsycINFO (via ProQuest)**	
S1	(mindfulness OR mindful* OR yoga OR meditation OR "acceptance and commitment therapy") AND ("psychotic disorders" OR psychosis OR psychot* OR psychos* OR schizophrenia OR "schizophrenia spectrum disorders")	2,820
S2	su(Mindfulness) AND su(psychosis)	137
S3	S1 OR S2 MeSH OR free text	2,818
S4	RCT (Clinical trials due NO RCT) & 1990-2020	152
	**The Cochrane Library (Wiley Online Library)**	
S1	'MeSH descriptor: [Psychotic Disorders] explode all trees'	3,104
S2	'MeSH descriptor: [Mindfulness] explode all trees'	1,037
S3	mindfulness OR mindful* OR yoga OR meditation OR "acceptance and commitment therapy"	12,073
S4	"psychotic disorders" OR psychosis OR psychot* OR psychos* OR schizophrenia OR "schizophrenia spectrum disorders"	61,717
S5	#3 AND #4	2,411
S6	#1 AND #2	10
S7	#5 OR #6	2,411
S8	"randomized controlled trial" OR RCT OR "randomized controlled trial"	930,086
S9	#7 AND #8	1,689
S10	1990 - 2020	1,540
**Hand search**	International Standard Randomised-Controlled Trial Number Register (ISRCTN)	25
	Clinicaltrials.gov	1
	National Center for Complementary and Alternative Medicine (nccam.nih.gov/clinicaltrials/alltrials.htm)	0
	WHO International Clinical Trial Registration Platform Search Portal	0
	Australian and New Zealand Clinical Trial Registry	5
S1	CNKI 中国知网<br>精神分裂症*(阴性症状+阳性症状+生活质量) AND 正念	225
S2	Extension of synonym (同义词扩展)	251
S3	1990 - 2020	203
	**Total**	**3,685**

For Peer Review Only

Appendix C Summary of participants and interventions characteristics

	Participants		Intervention			Control	Baseline demographics		Point of assessment
Study	Total n	Proportion with psychosis, %	Treatment protocol	Treatment duration	Home practice arrangement		Age, years: mean (s.d. or range)	Proportion female, %	
Behere et al. (2011)	91	100	Yoga with medication	One month	Keep a log for 2 months	Exercise or waitlist	Yoga : 31.3 (9.3)<br>Exercise: 30.2 (8.0)<br>Waitlist : 33.6 (9.9)	28.8	Baseline<br>2nd month<br>4th month
Budak & Yilmaz (2019)	50	100	Group yoga practice	Every weekday for 8 weeks, 50-55 mins	CDs yoga videos after 8 weeks	TAU	18-61<br>< 50: 90%<br>> 50: 6%	46	Pre- and Post-treatment
Caponnetto et al. (2019)	30	100	Yoga exercise plus standard rehabilitation (SRT)	12 weekly 1.5 hours	Not reported	SRT	Yoga+SRT: 36.9 (9.1)<br>SRT: 37.2 (9.5)	50	Pre- and Post-treatment
Chadwick et al. (2009)	21	100	Group-based mindfulness	5 weeks Twice per week	5 weeks with CDs	Waitlist	41.6 (8.1)	Not reported	Baseline<br>10 weeks
Chadwick et al. (2016)	108	100	Group Person-centered cognitive therapy (PBCT) plus TAU	12-weekly 1.5 hours	Non-compulsory 10-min CD	TAU	PBCT: 42 (18-65)<br>TAU : 42 (19-59)	50	Baseline<br>4th month<br>10th month
Chien et al. (2017)	342	100	Mindfulness-based psychoeducation group (MBPEG)	24-week biweekly 2 hours session	Not reported	Conventional psychoeduca-tion (CPEG) or TAU	25.1 (6.8)	36.8	Baseline<br>1 week<br>6 months<br>12 months<br>24 months
Chien et al. (2019)	180	100	Mindfulness-based psycho-education programme (MPGP)	6-month 12 2-hour session biweekly	Homework practice	Psychoeducat-ion programme TAU	25-28 (6.1-7.8)	44.44	Baseline<br>1 week<br>9 months<br>18 months
Davis et al. (2015)	34	100	Open group - Mindfulness intervention for rehabilitation and recovery in schizophrenia (MIRRORS)	16 weeks twice-weekly 75-min	Daily 20-30 minutes guided meditation	Intensive support	MIRRORS: 53.2 (6.1)<br>Support: 50.1 (10.6)	2.9	Baseline<br>8 weeks<br>16 weeks<br>24 weeks

For Peer Review Only

	Participants		Intervention			Control	Baseline demographics		Point of assessment
Study	Total n	Proportion with psychosis, %	Treatment protocol	Treatment duration	Home practice arrangement		Age, years: mean (s.d. or range)	Proportion female, %	
Duraiswamy et al. (2007)	61	100	Yoga therapy (YT)	3 weeks<br>5 days per week<br>1 hour/day	Self-practice for 3 months	Physical exercise (PT)	YT: 32.53(7.9)<br>PT: 31.30(7.9)	31.1	Baseline<br>4 months
Gumley et al. (2017)	29	100	Individual ACT for depression after psychosis (ACTdp) plus standard care (SC)	5-months	Not reported	SC alone	46.5 (9.0)	34.5	Baseline<br>5 months<br>10 months
Huang, Qin & Li (2017)	60	100	MI plus TAU	8 1.5-hour weekly sessions	Not reported	TAU plus psychoeduca-tion	MI: 38 (7.3)<br>TAU: 38.9 (7.6)	45	Pre- and Post-treatment
Ikai et al. (2013)	49	100	Yoga therapy	8-weekly<br>1- hour	Not reported	Regular day-care	Yoga : 54.8(9.0)<br>Control: 51.5(15.1)	34.7	Baseline<br>8 weeks
Ikai et al. (2014)	50	100	Hatha yoga plus regular treatment	8 weekly<br>1-hour	Not reported	Regular treatment & day care	Yoga : 53.5(9.9)<br>Control: 48.2(12.3)	34	Baseline<br>8 weeks
Ikai et al. (2017)	56	87.5	Chair yoga plus TAU	12-week twice weekly- 20 mins session	Not reported	TAU	55.3(13.7)	40	Baseline<br>6 weeks
Lam et al. (2020)	46	100	Mindfulness-based psychoeducation programme (MBPP)	8-week 1.5 hours	Daily MP3 guided mindfulness practice	TAU	Over 45	76.1	Baseline<br>Post-treatment<br>3 months
Langer et al. (2012)	18	100	MBCT	8 1-hour sessions	A CD on body scan, sitting meditation & homework forms to complete	Wait list	MBCT : 34.7 (8.2)<br>Control: 33.9 (10.7)	48.9	Pre- and Post-intervention
Lee (2019)	60	100	Mindfulness-based interventions (MBI)	8 1.5-hour session	Homework assignment	TAU	MBI: 54.43 (6.32)<br>TAU: 51.15 (6.32)	Not reported	Baseline<br>Post-course<br>3-month
Lin et al. (2015)	140	100	Yoga or aerobic	12 weeks<br>3 1-hour sessions per week	Not reported	Waitlist	Yoga :23.8 (6.8)<br>Aerobic:24.6 (7.9)<br>Waitlist: 25.3 (8.1)	100	Baseline<br>12 weeks<br>18 months

For Peer Review Only

	Participants		Intervention			Control	Baseline demographics		Point of assessment
Study	Total n	Proportion with psychosis, %	Treatment protocol	Treatment duration	Home practice arrangement		Age, years: mean (s.d. or range)	Proportion female, %	
Lopez-Navarro et al. (2015)	44	93.2	Integrated rehabilitation treatment (IRT) plus MI	26 1-hour weekly sessions	An audio tape with the guidance instructions	IRT	38.44 (8.06)	18.2	Pre- and Post-treatment
Lopez-Navarro et al. (2020)	52	92.3	Mindfulness group session	26-week 1 hour IRT plus MBI	An audio tape with the guidance instructions	IRT	IRT+MBI: 39.42 (8.63)<br>IRT: 40.15 (9.38)	11	Pre- and Post-treatment
MacDougall et al. (2018)	21	85.7	Group Mindfulness ambassador program (MAP)	12 weekly 1-hour sessions	Take-away assignments	TAU	23.71	23.5	Pre- and Post-treatment
Manjunath et al. (2013)	88	100	Yoga	2 weeks<br>1 hour daily	4 weeks self practice	Physical exercise	Yoga : 31.7 (8.8)<br>Exercise: 31.1 (7.8)	44.3	Baseline<br>2 weeks<br>6 weeks
Paikkatt et al. (2015)	30	100	Yoga plus medication	1 month (except holiday)<br>1.5 hours per day	Not repored	Medication	20-50	0	Pre- and Post-treatment
Shawyer et al. (2012)	43	93	Treatment of Resistant Command Hallucinations (TORCH)<br>(CBT plus ACT)	6 months<br>15 weekly sessions of 50 min plus 2 follow-up sessions	Audiotaped instructions	Befriending or waitlist	TORCH: 40.0 (8.5)<br>Befriending: 39.6 (11.4)	44.2	Baseline<br>6 months
Shawyer et al. (2017)	96	100	ACT	3 months<br>8 weekly or biweekly 50-min sessions	CD for review	Befriending	ACT: 35.6 (15.3)<br>Befriending: 33.0 (8.5)	38.5	Baseline<br>Post-therapy<br>6 months
Spidel et al. (2018)	50	73	ACT plus TAU	8 sessions<br>70-75 min	Not reported	TAU	40.4 (19-64)	52	Baseline<br>3th month
Varambally et al. (2012)	120	100	Yogasana	One month<br>Daily for 25 45-min sessions	A log book for record	Exercise or waitlist	Yogasana:32.8 (10.0)<br>Exercise:30.6 (7.3)<br>Waitlist : 33.6 (9.5)	30.3	Baseline<br>4th month
Visceglia & Lewis (2011)	18	100	Yoga therapy (YT)	8 weeks<br>45-mins twice weekly	Not reported	Waitlist (WT)	42 (13.5)<br>YT: 37.4 (13.73)<br>WT: 48.13 (11.24)	33.3	Baseline<br>8 weeks

For Peer Review Only

	Participants		Intervention			Control	Baseline demographics		Point of assessment
Study	Total n	Proportion with psychosis, %	Treatment protocol	Treatment duration	Home practice arrangement		Age, years: mean (s.d. or range)	Proportion female, %	
Wang (2018)	60	100	Mindfulness training plus TAU	8 weekly 2 hours	Self-practice 30- min daily	TAU	Mindfulness: 35.6 (9.2)<br>TAU: 36.9 (8.8)	48.33	Pre- and Post-treatment
White et al. (2011)	27	89.9	ACT plus TAU	10 1-hour sessions	Not reported	TAU alone	ACT: 33.57 (8.63)<br>TAU: 34.54 (10.97)	22.2	Baseline<br>3 months
Yang & Zhu (2019)	72	100	Mindfulness-based cognitive therapy plus TAU	From admission to discharge Monday to Saturday, am and pm	8-week 20-30-min guided practice daily	Routine health education and psychologic-al intervention plus TAU	Mindfulness: 42.2 (13.6)<br>Control: 43.5 (12.8)	41.67	Pre- and Post-treatment

n, number; s.d., standard deviation; TAU, treatment-as-usual; MI, mindfulness intervention; MBI, mindfulness-based intervention; CBT, cognitive-behavioral therapy; ACT, acceptance-and- commitment therapy;

For Peer Review Only

Appendix B Summary of study characteristics

Study	Country	Primary outcome measures (Name of measure)	Secondary outcome measures (Name of measure)	Treatment setting	Attrition %
Behere et al. (2011)	India	Psychotic symptoms (PANSS)<br>Emotions recognition ability (TRACS)	Socio-occupational functioning (SOFS)	Out-patient	Yoga: 20.5<br>Exercise: 45.1<br>Waitlist: 13.5
Budak & Yilmaz (2019)	Turkey	Insight into illness/treatment (BIS)<br>Medication adherence (MMAS)	NA	Community	Not reported
Caponnetto et al. (2019)	Italy	Affective symptoms (PSS)	QOL (EQ-5D)<br>Psychotic symptoms (PANSS)<br>Limits and resources (Mini-ICFAPP)	In-patient	Not reported
Chadwick et al. (2009)	England	Clincal functioning (CORE)	Responses to mindfulness (SMQ)<br>Auditory hallucination and delusion (PSYRATS)<br>Mindful responses to voices (SMVQ)<br>Beliefs about voices (BAVQ-r)<br>Rank eight therapeutic factors	Out-patient	18.2
Chadwick et al. (2016)	England	Clincal functioning (CORE-OM)	Auditory hallucination (PSYRATS: AHS)<br>Depression and anxiety (HADS)<br>Recovery goals for CBTp (CHOICE)	Out-patient	14 (at 4-months)<br>24 (at 10 months)
Chien et al. (2017)	Hong Kong<br>Mainland China<br>Taiwan	Average number and length of re-hospitalizations<br>Psychotic symptoms (PANSS)	Functioning (SLOF)<br>Insight into illness/treatment (ITAQ)	Community treatment facilities	19.3<br>10 attended < 4 sessions
Chien et al. (2019)	Hong Kong<br>China	Functioning (SLOF)	Insight into illness/treatment (ITAQ)<br>Psychotic symptoms (PANSS)<br>Mindfulness skills (FFMQ)	Out-patient	9.38
Davis et al. (2015)	USA	Work performance (WBI)	Number of hours worked weekly<br>Psychotic symptoms (PANSS)<br>Process of change (CAS)<br>Treatment adherence (MFS)<br>Client satisfaction (CSQ-8)	Out-patient	16.7
Duraiswamy et al. (2007)	India	Psychotic symptoms (PANSS)	Socio-occupational functioning(SOFS)<br>Side-effects (SAS)<br>Involuntary movement (AIMS)<br>QOL (WHOQOL-BREF)	Out-patient and in-patient	26

For Peer Review Only

Study	Country	Primary outcome measures (Name of measure)	Secondary outcome measures (Name of measure)	Treatment setting	Attrition %
Gumley et al. (2017)	UK	Depression (CDSS)<br>Depression (BDI)	Minfulness skills (KIMS)<br>Psychological flexibility (AAQ-II)<br>Recovery (QPR)	Out-patient and in-patient	13.8
Huang, Qin & Li. (2017)	China	Insight into illness/treatment (ITAQ)	Psychotic symptoms (BPRS)<br>Functioning (NOSIE)	In-patient	Not reported
Ikai et al. (2013)	Japan	Posture stability (CSP)	Anteflexion in standing<br>Psychotic symptoms (PANSS)<br>Side-effects (DIEPSS)<br>Functioning (FACT-Sz)<br>QOL (EQ-5D)	Out-patient	8 (yoga)<br>12.5 (control)
Ikai et al. (2014)	Japan	Resilience (RS)	Blood and saliva synthesis<br>Psychotic symptoms (PANSS)<br>Side-effects (DIEPSS)<br>Functioning (FACT-Sz)<br>QOL (EQ-5D)	Out-patient	28
Ikai et al. (2017)	Japan	Posture stability (CSP)	Psychotic symptoms (PANSS)<br>Depression (MADRS)<br>Mania (YMRS)<br>Cognition (MMSE)<br>Side-effects (DIEPSS)<br>Problems on schizophrenia (TIP-Sz)<br>Functioning (GAF)<br>QOL (EQ-5D)<br>Fall risk (MFES)	In-patient	Not reported
Lam et al. (2020)	Hong Kong	Emotion regulation (ERQ)<br>Rumination (SRRS)	Affective symptoms (DASS-21)<br>Psychotic symptoms (C-PSYRATS)<br>Mindfulness skills (FFMQ-SF)<br>Negative symptoms (SANS)	Community	4.3
Langer et al. (2012)	Spain	Clinical functioning (CGI-SCH)	Psychological flexibility (AAQ-II)<br>Responsed to mindfulness (SMQ)	Community	27.8
Lee (2019)	Taiwan	Mindfulness skills (MAAS)	Depression (BDI-II)<br>Negative symptoms (SANS)<br>Psychotic symptoms (CMV-PANSS)	In- and Out-patient	20
Lin et al. (2015)	Hong Kong	Cognition (HKLLT)	Depression (CDS)<br>Psychotic symptoms (PANSS)<br>Hippocampal volume (HPC)<br>QOL (SF-36)<br>Medication compliance (MCR)<br>Body perception (FRS)<br>Fitness by VO2 max	Out-patient	21.7
Lopez-Navarro et al. (2015)	Spain	Psychological QOL (WHOQOL-BREF)	QOL (WHOQOL-BREF)<br>Psychotic symptoms (PANSS)<br>Mindfulness skills (MAAS)	Community	6.8

Email: IANN-peerreview@journals.tandf.co.uk URL: http://mc.manuscriptcentral.com/sann

For Peer Review Only

Study	Country	Primary outcome measures (Name of measure)	Secondary outcome measures (Name of measure)	Treatment setting	Attrition %
Lopez-Navarro et al. (2020)	Spain	Inhibitory control (SCWT)	Mindfulness skills (MAAS)<br>Psychotic symptoms (PANSS)	Community	Not reported
MacDougall et al. (2018)	Canada	Client satisfaction (CSQ-8)	Self-esteem (RSES)<br>Emotional state (POMS)<br>Social functioning (SFS)<br>Mindfulness skills (KIMS)<br>Positive symptoms (SAPS)<br>Negative symptom (SANS)	Out-patient	19
Manjunath et al. (2013)	India	Clinical functioning (CGIS)<br>Psychotic symptoms (PANSS)	Psychotic symptoms (subscale PANSS)<br>Depression (HDRS)<br>Side-effects (SAS)	In-patient	31.8
Paikkatt et al. (2015)	India	Psychotic symptoms (PANSS)	NA	In-patient	6.7
Shawyer et al. (2012)	Australia	hallucinations (0-7 scale)	Psychotic symptoms (PANSS)<br>Auditory hallucination (PSYRATS: AHS)<br>Functioning (GAF)<br>QOL (QOL-LESQ)<br>Client satisfaction (CSQ-8)<br>Voices acceptance (VAAS)<br>Beliefs about the voices (BAVQ-R)<br>Insight into illness/treatment (IS)<br>Recovery (RSQ)	Community	6.8
Shawyer et al. (2017)	Australia	Psychotic symptoms (PANSS)	Auditory hallucination and delusion (PSYRATS)<br>Delusions (PDI)<br>Psychosocial functioning (SFS)<br>Psychological flexibility (AAQ II)<br>Voices acceptance (VAAS)<br>Recovery (RSQ)<br>Client satisfaction (CSQ)<br>Service utilization	Community	11.5
Spidel et al. (2018)	Canada	Emotion Regulation (CERQ)	Psychotic symptoms (BPRS-E)<br>Trauma Symptom (TSC-40)<br>Anxiety (GAD-7)<br>Service Engagement (SES)	Community	Unclear
Varambally et al. (2012)	India	Psychotic symptoms (PANSS)	Socio-occupational functioning (SOFS)<br>Side-effects (EPS)	Out-patient	20.2
Visceglia & Lewis (2011)	New York	Psychotic symptoms (PANSS)	QOL (WHOQOL-BREF)	In-patient	Not reported
Wang (2018)	China	Cognitive function (WCST)	NA	In-patient	Not reported
White et al. (2011)	England	Anxiety and depression (HDRS)<br>Psychotic symptoms (PANSS)	Psychological flexibility (AAQ II)<br>Mindfulness skills (KIMS)<br>Therapeutic alliance (WAI-SR)	Community	11.1

For Peer Review Only

Study	Country	Primary outcome measures (Name of measure)	Secondary outcome measures (Name of measure)	Treatment setting	Attrition %
Yang & Zhu (2019)	China	Self-compassion (SCS-C)<br>Insight into illness/treatment (SSMI-C)	Emotional state (HAMA)<br>Emotional state (HAMD)<br>QOL (WHO-QOL-BREF)	In-patient	Not reported

AAQII, Acceptance and Action Scale; AIMS, Abnormal Involuntary Movements Scale; BAVQ-r, Beliefs about Voices Questionnaire revised; BDI, Beck Depression Inventory; BIS, Birchwood Insight Scale; BPRS, The Brief Psychiatric Rating Scale; BPRS-E, The Brief Psychiatric Rating Scale-Expanded; CAS, The Change Assessment Scale; CDS, Calgary Depression Scale; CDSS, Calgary Depression Scale for Schizophrenia; CERQ, Cognitive Emotion Regulation Questionnaire; CGIS, The Clinical Global Impression Severity; CGI-SCH, Clinical Global Impression-Schizophrenia Scale; CHOICE, Choice of Outcome in CBT for Psychosis; CMV-PANSS, Chinese Mandarin version of the positive and negative syndrome scale; CORE, Clinical Outcomes in Routine Evaluation; CORE-OM, Clinical Outcomes in Routine Evaluation-outcome measure; CSP, The Clinical Stabilometric Platform; CSQ-8, The Client Satisfaction Questionnaire; DASS-21, Depression Anxiety Stress Scale; DIEPSS, The Drug Induced Extrapyramidal Symptoms Scale; EPS, Extra-pyramidal symptoms; EQ-5D, the EuroQol-5 dimensions; ERQ, Emotion Regulation Questionnaire; EuroQoLVAS, EuroQol Visual Analogue Scale; FACT-Sz, the Functional Assessment for Comprehensive Treatment of Schizophrenia; FFMQ, Five Facet Mindfulness Questionnaire; FFMQ-SF, Five Facet Mindfulness Questionnaire-short form; FRS, Figure Rating scale; GAD-7, The Generalized Anxiety Disorder Scale – 7; GAF, Global Assessment of Functioning; HADS, Hospital Anxiety and Depression Scale; HAMA, Hamilton Rating Scale for Anxiety; HAMD, Hamilton Rating Scale for Depression; HDRS, Hamilton depression rating scale; HKLLT, Hong Kong list learning test; HPC, Hippocampus; IS, The Insight Scale; ITAQ, Insight and Treatment Attitudes Questionnaire; KIMS, Kentucky Inventory of Mindfulness Skills; MAAS, Mindfulness Attention Awareness Scale; MADRS, the Montgomery-Åsberg Depression Rating Scale; MCR, medication compliance rating; MFES, The Modified Falls Efficacy Scale; MFS, The Mindfulness Fidelity Scale; The Mini-ICFAPP; MMSE, Mini Mental State Examination; MMAS, Morisky Medication Adherence Scale; NOSIE, Nurses Observation Scale For Inpatient Evaluation; PANSS, The Positive and Negative Syndrome Scale; PDI, Peters Delusions Inventory; POMS, The Profile of Mood States; PSS, The Perceived Stress Scale; PSYRATS, Psychiatric Symptom Rating Scale; PSYRATS-AHS, Psychiatric Symptom Rating Scale-Auditory Hallucinations Scale; QOL-LESQ, The Quality of Life Enjoyment and Satisfaction Questionnaire; QPR, Process of Recovery; RS, Resilience Scale; RSES, Self-Esteem Scale; RSQ, Recovery Style Questionnaire; SANS, Scale for Assessment of Negative Symptoms; SAPS, Scale for Assessment of Positive Symptoms; SAS, Simpson angus scale for extrapyramidal side effects; SCWT, Stroop Color World Test; SCS-C, Self-compassion Scale-Chinese; SES, The Service Engagement Scale; SF-36, SF-36 Health Survey; SFS, Social Functioning Scale; SLOF, Specific Levels of Functioning Scale; SMQ, Southampton Mindfulness Questionnaire; SMVQ, Southampton Mindfulness Voices Questionnaire; SOFS, Socio-Occupational Functioning Scale; SRRS, Short Ruminative Response Scale; SSMI-C, Stigma Scale for Mental Illness-Chinese; TIP-Sz, The Targeted Inventory on Problems in Schizophrenia; TRACS, TRENDS Accuracy Score; TSC-40, The Trauma Symptom Checklist-40; VAAS, Voices Acceptance and Action Scale; WAI-SR, Working Alliance Inventory; WBI, The Work Behaviour Inventory; WCST, Winsconsin Card Sorting Test; WHOQOL-BREF, World Health Organization Quality of Life; YMRS, The Young Mania Rating Scale

For Peer Review Only

Appendix H Summary of findings

Correlate outcomes (number of studies)	Studies included	n	✓= primary Outcome measures	Main findings	Overall Quality (GRADE)
Psychotic symptoms (22)	Behere et al. (2011)<br>Caponnetto et al. (2019)<br>Chien et al. (2017)<br>Chien et al. (2019)<br>Duraiswamy et al. (2007)<br>Huang, Qin & Li (2017)<br>Ikai et al. (2013)<br>Ikai et al. (2014)<br>Ikai et al. (2017)<br>Lee (2019)<br>Lin et al. (2015)<br>Lopez-Navarro et al. (2015)<br>Lopez-Navarro et al. (2020)<br>MacDougall et al. (2018)<br>Manjunath et al. (2013)<br>Paikkatt et al. (2015)<br>Shawyer et al. (2012)<br>Shawyer et al. (2017)<br>Spidel et al. (2018)<br>Varambally et al. (2012)<br>Visceglia & Lewis (2011)<br>White et al. (2011)	1,632	PANSS ✓<br>PANSS<br>PANSS ✓<br>PANSS<br>PANSS ✓<br>BPRS<br>PANSS<br>PANSS<br>PANSS<br>CMV-PANSS<br>PANSS<br>PANSS<br>PANSS<br>SAPS; SANS<br>PANSS ✓<br>PANSS ✓<br>PANSS<br>PANSS ✓<br>BPRS-E<br>PANSS ✓<br>PANSS ✓<br>PANSS✓	May improve slightly on the psychotic symptoms of all studies.<br><br>Significant difference on reduction of psychotic symptoms when Yoga and PsychoED as the interventions	***LOW***
Affective symptoms (11)	Caponnetto et al. (2019)<br>Chadwick et al. (2016)<br>Gumley et al. (2017)<br>Lam et al. (2020)<br>Lee (2019)<br>Lin et al. (2015)<br>MacDougall et al. (2018)<br>Manjunath et al. (2013)<br>Spidel et al. (2018)<br>White et al. (2011)<br>Yang & Zhu (2019)	641	PSS ✓<br>HADS<br>CDSS✓; BDI-II ✓<br>DASS-21<br>BDI-II<br>CDS<br>POMS<br>HDRS<br>GAD-7<br>HADS ✓<br>HAMA; HAMD	May have little or no difference in reducing affective symptoms, but ACT comparatively significant as compared with controls	***Very LOW***
Quality of life (10)	Caponnetto et al. (2019)<br>Duraiswamy et al. (2007)<br>Ikai et al. (2013)<br>Ikai et al. (2014)<br>Ikai et al. (2017)<br>Lin et al. (2015)<br>Lopez-Navarro et al. (2015)<br>Shawyer et al. (2012)<br>Visceglia & Lewis (2011)<br>Yang & Zhu (2019)	547	EuroQoL VAS<br>WHOQOL-BREF<br>EQ-5D<br>EQ-5D<br>EQ-5D<br>QOL (SF-36)<br>WHOQOL-BREF ✓<br>QOL-LESQ<br>WHOQOL-BREF<br>WHOQOL-BREF	Probably little or no difference in improving QoL though Yoga and MI comparatively more significant than other interventions	***MODERATE***
Socio-occupational Functioning (9)	Behere et al. (2011)<br>Chien et al. (2017)<br>Chien et al. (2019)<br>Duraiswamy et al. (2007)<br>Ikai et al. (2017)<br>McDougall et al. (2018)<br>Shawyer et al. (2012)<br>Shawyer et al. (2017)<br>Varambally et al. (2012)	968	SOFS<br>SLOF<br>SLOF✓<br>SOFS<br>GAF<br>SFS<br>GAF<br>SFS<br>SOFS	Uncertain whether MI improves functioning<br><br>However, functioning strongly and significantly associated with PsychoED	***VERY LOW***

For Peer Review Only

Correlate outcomes (number of studies)	Studies included	n	✓= primary Outcome measures	Main findings	Overall Quality (GRADE)
Mindfulness skills (8)	Chien et al. (2019)<br>Gumely et al. (2017)<br>Lam et al. (2020)<br>Lee (2019)<br>Lopez-Navarro et al. (2015)<br>Lopez-Navarro et al. (2020)<br>MacDougall et al. (2018)<br>White et al. (2011)	433	FFMQ<br>KIMS<br>FFMQ-SF<br>MAAS ✓<br>MAAS<br>MAAS<br>KIMS<br>KIMS	Probably little or no difference whether MBIs makes improvement<br><br>Significant difference in increasing mindfulness skills when MI and PsychoED as the interventions	***MODERATE***
Clinical functioning (7)	Chadwick et al. (2009)<br>Chadwick et al. (2016)<br>Langer et al. (2012)<br>Huang, Qin & Li (2017)<br>Ikai et al. (2013)<br>Ikai et al. (2014)<br>Manjunath et al. (2013)	365	CORE ✓<br>CORE-OM ✓<br>CGI-SCH ✓<br>NOSIE<br>FACT-Sz<br>FACT-Sz<br>CGIS ✓	Probably little or no difference whether MBIs makes improvement	***VERY LOW***
Insight into illness/ treatment (6)	Budak and Yilmaz (2019)<br>Chien et al. (2017)<br>Chien et al (2019)<br>Huang, Qin & Li (2017)<br>Shawyer et al. (2012)<br>Yang & Zhu (2019)	736	BIS<br>ITAQ<br>ITAQ<br>ITQA ✓<br>IS<br>SSMI-C✓	Strongly and significantly when associated with MBIs, while Yoga and PsychoED comparatively more significant than other interventions, but little or no difference in increasing insight into illness	***VERY LOW***
Auditory hallucination and delusion (5)	Chadwick et al. (2009)<br>Chadwick et al. (2016)<br>Lam et al. (2020)<br>Shawyer et al. (2012)<br>Shawyer et al. (2017)	315	PSYRATS<br>PSTRATS-AHS<br>C-PSYRATS<br>PSYRATS<br>PSYRATS	Probably little or no difference whether MBIs makes improvement	***MODERATE***
Side-effects (5)	Duraiswamy et al. (2007)<br>Ikai et al. (2013)<br>Ikai et al. (2014)<br>Ikai et al. (2017)<br>Manjunath et al. (2013)	304	SAS<br>DIEPSS<br>DIEPSS<br>DIEPSS<br>SAS	Probably little or no difference in improving side-effects	***MODERATE***
Psychological flexibility (4)	Gumley et al. (2007)<br>Langer et al. (2012)<br>Shawyer et al. (2017)<br>White et al. (2011)	170	AAQII<br>AAQII<br>AAQII<br>AAQII	Probably little or no difference whether MBIs makes improvement	***MODERATE***
Client satisfaction (3)	Davis et al. (2015)<br>Shawyer et al. (2012)<br>Shawyer et al. (2017)	173	CSQ-8<br>CSQ-8<br>CSQ-8✓	May have little or no difference in increasing recovery	***Moderate***
Emotion regulation (3)	Behere et al. (2011)<br>Lam et al. (2020)<br>MacDougall et al. (2018)	162	TRAC ✓<br>ERQ ✓<br>CERQ ✓	Probably little or no difference whether MBIs makes improvement	***Very Low***

For Peer Review Only

Appendix F Effect sizes and heterogeneity statistics analyzed by groups of studies

Outcome[a]	Studies group	N[b]	Effect Size (g)	95% CI	Q	I² (%)	τ^2	Different in Subgroup
Psychotic symptoms		22	-0.48*	[-0.95 , -0.01]	300.20***	93.00	1.16	
Type of intervention					18.18***	83.50		<0.001
	Yoga	11	-0.40***	[-0.66 , -0.14]	19.78*	49.00	0.09	
	ACT	4	-0.27	[-0.82 , 0.29]	9.86*	70.00	0.22	
	MI	5	0.27	[-0.88 , 1.42]	61.84***	94.00	1.59	
	PsychoED	2	-2.78***	[-3.92 , -1.64]	11.45***	91.00	0.62	
Nature of outcome					0.57	0.00		0.45
	Primary outcome	10	-0.68*	[-1.27 , -0.08]	106.83***	92.00	0.81	
	Secondary outcomes	12	-0.31	[-1.06 , 0.44]	185.50***	94.00	1.64	
Time Effects[c]					4.49*	77.70		0.03
	Long-term	3	-1.98**	[-3.46 , -0.50]	51.56***	96.00	1.64	
	Short-term	21	-0.33	[-0.68 , 0.01]	156.18***	87.00	0.56	
Age group					4.06	26.20		0.25
	20 - 50	3	-1.58	[-3.58. , 0.41]	40.90***	95.00	2.94	
	30 - 50	13	-0.36***	[-0.62 , -0.11]	28.93**	59.00	0.13	
	30	2	-1.28	[-3.17 , 0.61]	28.67***	97.00	1.79	
	Over 50	4	0.49	[-0.79 , 1.76]	51.76***	94.00	1.58	
Affective symptoms		11	-0.44*	[-0.83 , -0.05]	48.79**	80.00	0.34	
Type of intervention					2.59	0.00		0.46
	Yoga	3	-0.16	[-0.84 , 0.53]	9.26**	89.00	0.50	
	ACT	3	-0.64**	[-1.15 , -0.12]	3.66	45.00	0.09	
	MI	4	-0.59	[-1.51 , 0.32]	28.47***	89.00	0.76	
	PsychoED	1	-0.08	[-0.66 , 0.50]				
Quality of life		10	0.38***	[0.19 , 0.56]	15.12	40.00	0.06	
Type of intervention					9.07**	77.90		0.01
	Yoga	7	0.49***	[0.26 , 0.72]	5.03	0.00	0.00	
	ACT	1	-0.60	[-1.27 , 0.07]				
	MI	2	0.38*	[0.00 , 0.75]				
Socio-occupational functioning		9	0.58	[-0.40 , 1.56]	253.66***	97.00	2.17	
Type of intervention					225.43***	98.70		<0.001
	Yoga	4	-0.06	[-0.35 , 0.23]	3.52	15.00	0.01	
	ACT	2	-0.19	[-0.65 , 0.27]	1.54	35.00	0.04	
	MI	1	0.16	[-0.80 , 1.11]				
	PsychoED	2	2.90***	[2.59 , 3.21]	0.00	0.00	0.00	
Mindfulness skills		8	0.45***	[0.23 , 0.68]	7.90	11.00	0.01	
Type of intervention					2.91	0.00		0.41
	Yoga	1	-0.01	[-0.57 , 0.56]				
	ACT	2	0.58	[-0.02 , 1.19]	1.26	20.00	0.04	
	MI	3	0.41*	[0.04 , 0.78]	1.00	0.00	0.00	
	PsychoED	2	0.58**	[0.12 , 1.04]	1.82	45.00	0.05	
Clinical functioning		7	0.73	[-0.46 , 1.91]	130.81***	95.00	2.37	
Type of intervention					1.29	22.40		0.26
	Yoga	3	-0.01	[-1.31 , 1.10]	26.94***	93.00	1.05	
	MI	4	1.53	[-1.01 , 4.07]	102.45***	97.00	6.44	
Insight into illness/ treatment		5	1.35**	[0.32 , 2.39]	126.46***	96.00	1.57	
Type of intervention					24.29***	87.60		<0.001
	Yoga	1	2.92***	[2.10 , 3.73]				
	ACT	1	0.30	[-0.35 , 0.95]				
	MI	2	1.30	[-3.01 , 5.61]	81.29***	99.00	9.55	
	PsychoED	2	1.28***	[0.98 , 1.58]	1.57	36.00	0.02	

[a] Excluding outcomes that displayed homogeneity variation among the studies.

[b] Statistics for heterogeneity are not shown in studies group with one study.

[c] Two studies, namely, Chien et al (2017) and Lin et al (2015), were included in short-term and long-term analyses

* P < 0.05; ** P<0.01; *** P<0.001

For Peer Review Only

Appendix D GRADEpro and risk of bias (RoB) (2.0) assessment of outcomes

Author(s): Behere et al. (2011); Budak & Yilmaz (2019); Caponnetto et al. (2019); Chadwick et a.l (2009); Chadwick et al. (2016); Chien et al. (2017); Chien et al. (2019); Davis et al. (2015); Duraiswamy et al. (2007); Gumley et al. (2017); Huang, Qin & Li (2017); Ikai et al. (2013); Ikai et al. (2014); Ikai et al. (2017); Lam et al. (2020); Langer et al. (2012); Lee (2019); Lin et al. (2015); Lopez-Navarro et al. (2015); Lopez-Navarro et al. (2020); MacDougall et al. (2018); Manjunath et al. (2013); Paikkatt et al. (2015); Shawyer et al. (2012); Shawyer et al. (2017); Spidel et al. (2018); Varambally et al. (2012); Visceglia & Lewis (2011); Wang (2018); White et al. (2011); Yang & Zhu (2019).

Question: How is the effectiveness of pure MBIs on various outcomes for people with psychosis when compared with usual treatment and/or other psychosocial intervention?

Setting: Out-patient; in-patient and community treatment facilitates

Bibliography:

Behere, R. V., Arasappa, R., Jagannathan, A., Varambally, S., Venkatasubramanian, G., Thirthalli, J., Subbakrishna, D. K., Nagendra, H. R. & Gangadhar, B. N. (2011). Effect of yoga therapy on facial emotion recognition deficits, symptoms and functioning in patients with schizophrenia. *Acta Psychiatrica Scandinavica*, 123, 147-153.

Budak, F.K & Yilmaz, E. (2019). The effect of yoga on clinical insight and medication adherence in patients with schizophrenia - A randomized controlled trial. *European Journal of Integrative Medicine,* 30**,** 100949. https://doi.org/10.1016/j.eujim.2019.100949

Caponnetto, P., Auditore, R., Maglia, M., Pipitone, S., and Inguscio, L. (2019). Psychological wellness, yoga and quality of life in patients affected by schizophrenia spectrum disorders: A pilot study. *Mental illness*, *11*(1), 8003. https://doi.org/10.4081/mi.2019.8003

Chadwick, P., Hughes, S., Russell, D., Russell, I., and Dagnan, D. (2009). Mindfulness Groups for Distressing Voices and Paranoia: A Replication and Randomized Feasibility Trial. *Behavioural and Cognitive Psychotherapy*, 37(4), 403-412. doi:10.1017/S1352465809990166.

Chadwick, P., Strauss, C., Jones, A.M., Kingdon, D., Ellett, L., Dannahy, L. & Hayward, M. (2016). Group mindfulness-based intervention for distressing voices: A pragmatic randomised controlled trial. *Schizophrenia Research*, 175, 168-173. https://doi.org/10.1016/j.schres.2016.04.001.

Chien, W. T., Bressington, D., Yip, A. & Karatzias, T. (2017). An international multi-site, randomized controlled trial of a mindfulness-based psychoeducation group programme for people with schizophrenia. *Psychological Medicine*, 47(12), 2081–2096. doi: 10.1017/S0033291717000526.

Chien, W.T., Cheng, H.Y., McMaster, T.W., Yip, A.L.K. & Wong, J.C.L. (2019). Effectiveness of a mindfulness-based psychoeducation group programme for early-stage schizophrenia: An 18-month randomised controlled trial. *Schizophr Res.,* 212 (140-149). doi: 10.1016/j.schres.2019.07.053.

Davis, L. W., Lysaker, P. H., Kristeller, J. L., Salyers, M. P., Kovach, A. C. & Woller, S. (2015). Effect of mindfulness on vocational rehabilitation outcomes in stable phase schizophrenia. *Psychol Serv*., 12, 303-12.

Duraiswamy, G., Thirthalli, J., Nagendra, H.R. & Gangadhar, B.N. (2007). Yoga therapy as an add-on treatment in the management of patients with schizophrenia - a randomized controlled trial. *Acta Psychiatrica Scandinavica*, 116, 226–232.

Gumley, A., White, R., Briggs, A., Ford, I., Barry, S., Stewart, C., Beedie, S., Mctaggart, J., Clarke, C., Macleod, R. et al. (2017). A parallel group randomised open blinded evaluation of Acceptance and Commitment Therapy for depression after psychosis: pilot trial outcomes (ADAPT). *Schizophrenia research,* 183**,** 143-150. doi: 10.1016/j.schres.2016.11.026.

Huang, Qin & Li (2017). 正念訓練干預對住院精神分裂症患者的影響. *Today Nurse*, July, 7, 108-109.

For Peer Review Only

Ikai, S., Uchida, H., Suzuki, T., Tsunoda, K., Mimura, M. & Fujii, Y. (2013). Effects of yoga therapy on postural stability in patients with schizophrenia-spectrum disorders: A single-blind randomized controlled trial. *Journal of Psychiatric Research*, 47 (11), 1744-1750, doi: 10.1016/j.jpsychires.2013.07.017.

Ikai, S., Suzuki, T., Uchida, H., Saruta, J., Tsukinoki, K., Fujii, Y. & Mimura, M. (2014). Effects of weekly one-hour Hatha yoga therapy on resilience and stress levels in patients with schizophrenia-spectrum disorders: an eight-week randomized controlled trial. *Journal Of Alternative And Complementary Medicine,* 20**,** 823-830. doi: 10.1089/acm.2014.0205.20**,** pp. 823-830. doi: 10.1089/acm.2014.0205.

Ikai, S., Uchida, H., Mizuno, Y., Tani, H., Nagaoka, M., Tsunoda, K., Mimura, M. & Suzuki, T. (2017). Effects of chair yoga therapy on physical fitness in patients with psychiatric disorders: A 12-week single-blind randomized controlled trial. *J Psychiatr Res,* 94**,** 194-201. doi: 10.1016/j.jpsychires.2017.07.015.

Lam, A., Leung, S. F., Lin, J. J., & Chien, W. T. (2020). The Effectiveness of a Mindfulness-Based Psychoeducation Programme for Emotional Regulation in Individuals with Schizophrenia Spectrum Disorders: A Pilot Randomised Controlled Trial. *Neuropsychiatric disease and treatment*, *16*, 729–747. https://doi.org/10.2147/NDT.S231877

Langer, Á. I., Cangas, A. J., Salcedo, E. & Fuentes, B. (2012). Applying mindfulness therapy in a group of psychotic individuals: a controlled study. *Behavioural and Cognitive Psychotherapy*, 40(1), 105–109. doi: 10.1017/S1352465811000464.

Lee, K.H. (2019). A randomized controlled trial of mindfulness in patients with schizophrenia. *Psychiatry Res*., 275:137-142. doi: 10.1016/j.psychres.2019.02.079.

Lin, J., Chan, S. K., Lee, E. H., Chang, W. C., Tse, M. …. Chen, E.Y.H. (2015). Aerobic exercise and yoga improve neurocognitive function in women with early psychosis. *NPJ schizophrenia*, 1(0), 15047. https://dx.doi.org/10.1038/npjschz.2015.47.

Lopez-Navarro, E., Del Canto, C., Belber, M., Mayol, A., Fernandez-Alonso, O., Lluis, J., Munrar, E. & Chadwick, P. (2015). Mindfulness improves psychological quality of life in community-based patients with severe mental health problems: a pilot randomized clinical trial. *Schizophrenia Research*, 168, 530–536. https://doi.org/10.1016/j.schres.2015.08.016.

López-Navarro, E., Del Canto, C., Mayol, A., Fernández-Alonso, O., Reig, J. & Munar, E. (2020). Does mindfulness improve inhibitory control in psychotic disorders? A randomized controlled clinical trial. *International Journal of Clinical and Health Psychology,* 20**,** 192-199. https://doi.org/10.1016/j.ijchp.2020.07.002

MacDougall, A.G., Price, E., Vandermeer, M.R.J., Lloyd, C., Bird, R., Sethi, R., ….Norman, R.M.G. (2019). Youth-focused group mindfulness-based intervention in individuals with early psychosis: A randomized pilot feasibility study. *Early Interv Psychiatry,* 13(4):993-998. doi: 10.1111/eip.12753.

Manjunath, R. B., Varambally, S., Thirthalli, J., Basavaraddi, I. V. & Gangadhar, B. N. (2013). Efficacy of yoga as an add-on treatment for in-patients with functional psychotic disorder. *Indian Journal of Psychiatry*, 55(Suppl 3), 374–378. doi:10.4103/0019-5545.116314.

Paikkatt, B., Singh, A. R., Singh, P. K., Jahan, M. & Ranjan, J. K. (2015). Efficacy of Yoga therapy for the management of psychopathology of patients having chronic schizophrenia. *Indian Journal of Psychiatry*, 57(4), 355-360. doi: 10.4103/0019-5545.171837.

Shawyer, F., Farhall, J., Mackinnon, A., Trauer, T., Sims, E., Ratcliff, K., …. Copolov, D. (2012). A randomised controlled trial of acceptance-based cognitive behavioural therapy for command hallucinations in psychotic disorders. *Behaviour Research and Therapy*, 50(2), 110-121. https://doi.org/10.1016/j.brat.2011.11.007.

Shawyer, F., Farhall, J., Thomas, N., Hayes, S. C., Gallop, R., Copolov, D. & Castle, D. J. (2017). Acceptance and commitment therapy for psychosis: randomised controlled trial. *British Journal of Psychiatry*, 210(2), 140–148. doi: 10.1192/bjp.bp.116.182865.

Spidel, A., Lecomte, T., Kealy, D. & Daigneault, I. (2018). Acceptance and commitment therapy for psychosis and trauma: improvement in psychiatric symptoms, emotion regulation, and treatment compliance following a brief group intervention. *Psychology Psychotherapy: Theory, Research and Practice*, 91, 248-261. doi: 10.1111/papt.12159.

For Peer Review Only

Varambally, S., Gangadhar, B. N., Thirthalli, J., Jagannathan, A., Kumar, S., Venkatasubramanian, G., …Nagendra, H. R. (2012). Therapeutic efficacy of add-on yogasana intervention in stabilized outpatient schizophrenia: randomized controlled comparison with exercise and waitlist. *Indian journal of psychiatry,* 54**,** 227-232. doi: 10.4103/0019-5545.102414.
Visceglia, E. & Lewis, S. (2011). Yoga therapy as an adjunctive treatment for schizophrenia: a randomized, controlled pilot study. *The Journal of Alternative and Complementary Medicine*, 17 (7), 601–607. doi: 10.1089/acm.2010.0075.
Wang, Y.P. (2018). Study on the effect of mindfulness training intervention on the cognitive function of schizophrenia during consolidating period. *China Modern Doctor*, 56 (18), 64-67.
White. R., Gumley, A., McTaggart, J., Rattrie, L., McConville, D., Cleare, S. & Mitchell, G. (2011). A feasibility study of Acceptance and Commitment Therapy for emotional dysfunction following psychosis. *Behav Res Ther.* 49(12), 901-7. doi: 10.1016/j.brat.2011.09.003
Yang, Y.F. & Zhu, W.B. (2019). Effect of mindfulness-based cognitive therapy on self-pity and stigma of first-episode schizophrenia patients. China Modern Doctor, 57 (17), 91-94.

**Certainty assessment**							**№ of patients**		**Effect**	**Certainty**	**Importance**
**№ of studies**	**Study design**	**Risk of bias**	**Inconsistency**	**Indirectness**	**Imprecision**	**Other considerations**	**mindfulness-based interventions**	**comparators**	**Absolute (95% CI)**		
**Psychotic symptoms (assessed with: PANSS, BPRS)**											
22 #	randomised trials	not serious	very serious [a]	not serious	not serious	none	752	718	SMD **0.48 SD lower** (0.95 lower to 0.01 lower)	⊕⊕◯◯ LOW	CRITICAL
**Affective symptoms (assessed with: HADS, CDSS, BDI-II, CDS, HDRS, GAD-7)**											
11 #	randomised trials	serious [b]	serious [C]	not serious	serious [c]	none	297	276	SMD **0.44 SD lower** (0.83 lower to 0.05 lower)	⊕◯◯◯ VERY LOW	CRITICAL
**Quality of life (assessed with: WHOQOL-BREF, EQ-5D, QOL-SF 36, QOL-LESQ)**											
10 #	randomised trials	not serious	not serious	not serious	serious [c]	none	248	239	SMD **0.38 SD higher** (0.19 lower to 0.56 higher)	⊕⊕⊕◯ MODERATE	IMPORTANT

Certainty assessment							№ of patients		Effect	Certainty	Importance
**№ of studies**	**Study design**	**Risk of bias**	**Inconsistency**	**Indirectness**	**Imprecision**	**Other considerations**	**mindfulness-based interventions**	**comparators**	**Absolute (95% CI)**		
**Socio-occupational functioning (assessed with: SOFS, SLOF, GAF, SFS)**											
9	randomised trials	not serious	very serious [d]	not serious	very serious [c, e]	none	361	349	SMD **0.58 SD higher** (0.4 lower to 1.56 higher)	⊕◯◯◯ VERY LOW	IMPORTANT
**Mindfulness skills (assessed with: KIMS, MAAS, FFMQ, FFMQ-SF)**											
8	randomised trials	not serious	not serious	not serious	serious [c]	none	186	191	SMD **0.45 SD higher** (0.23 higher to 0.68 higher)	⊕⊕⊕◯ MODERATE	IMPORTANT
**Clinical functioning (assessed with: CORE, CORE-OM, NOSIE, FACT-SZ, CGI-SCH, CGIS)**											
7	randomised trials	serious [b]	serious [b]	not serious	serious [c]	none	188	188	SMD **0.73 SD higher** (0.46 lower to 1.91 higher)	⊕◯◯◯ VERY LOW	CRITICAL
**Insight into illness/treatment (assessed with: BIS, ITAQ, IS, SSMI-C)**											
6	randomised trials	serious [b]	very serious [a]	not serious	very serious [c,e]	none	359	359	SMD 1.**35 SD higher** (0.32 lower to 2.39 higher)	⊕◯◯◯ VERY LOW	IMPORTANT

For Peer Review Only

Certainty assessment							№ of patients		Effect	Certainty	Importance
**№ of studies**	**Study design**	**Risk of bias**	**Inconsistency**	**Indirectness**	**Imprecision**	**Other considerations**	**mindfulness-based interventions**	**comparators**	**Absolute (95% CI)**		
**Auditory hallucination and delusion (follow up: range 10 weeks to 6 months; assessed with: PSYRATS, PSTRATS-AHS, C-PSYRATS)**											
4	randomised trials	not serious	not serious	not serious	serious [c]	none	154	154	SMD **0.00 SD** (0.26 lower to 0.27 higher)	⨁⨁⨁◯ MODERATE	NOT IMPORTANT
**Side-effects (assessed with: SAS, DIEPSS)**											
5	randomised trials	not serious	not serious	not serious	serious [c]	none	134	122	SMD **0.08 SD lower** (0.49 lower to 0.32 higher)	⨁⨁⨁◯ MODERATE	IMPORTANT
**Psychological flexibility (assessed with: AAQII; Scale from: 10 to 70)**											
4	randomised trials	not serious	not serious	not serious	serious [c]	none	85	85	SMD **0.09 SD lower** (0.42 lower to 0.23 higher)	⨁⨁⨁◯ MODERATE	IMPORTANT
**Client satisfaction (follow up: range 8 weeks to 6 months; assessed with: CSQ-8; Scale from: 4 to 32)**											
3	randomised trials	not serious	not serious	not serious [c]	serious [c]	none	86	84	SMD **0.37 SD higher** (0.01 lower to 0.76 higher)	⨁⨁⨁◯ MODERATE	IMPORTANT

Email: IANN-peerreview@journals.tandf.co.uk URL: http://mc.manuscriptcentral.com/sann

Certainty assessment							№ of patients		Effect	Certainty	Importance
**№ of studies**	**Study design**	**Risk of bias**	**Inconsistency**	**Indirectness**	**Imprecision**	**Other considerations**	**mindfulness-based interventions**	**comparators**	**Absolute (95% CI)**		
**Emotion regulation (follow-up: mean 3 months; assessed with: TRAC, ERQ, CERQ)**											
3	Randomised trials	very serious [f]	not serious	not serious	serious[c]	publication bias strongly suspected	81	64	SMD **0.18 SD higher** (0.16 lower to 0.52 higher)	⨁◯◯◯ VERY LOW	IMPORTANT

CI: Confidence interval
SMD: Standardised mean difference
Funnel plot is only for outcomes with 10 studies or more

Explanations:
a. -2 (I^2=93)
b. More than 50% of studies sharing an outcome obtained at least one "High" risks
c. small sample size
d. -2 (I^2=97)
e. Wide CI
f. Study designs with serious limitations

For Peer Review Only
